# Prospective in (Primate) Dental Analysis through Tooth 3D Topographical Quantification

**DOI:** 10.1371/journal.pone.0066142

**Published:** 2013-06-24

**Authors:** Franck Guy, Florent Gouvard, Renaud Boistel, Adelaïde Euriat, Vincent Lazzari

**Affiliations:** Centre National de la Recherche Scientifique, Institut Ecologie et Environnement, UMR 7262 – iPHEP: Institut de Paléoprimatologie et Paléontologie Humaine, Evolution et Paléoenvironnements, Université de Poitiers, Faculté des Sciences, Poitiers, France; Museo Nazionale Preistorico Etnografico ‘L. Pigorini’, Italy

## Abstract

The occlusal morphology of the teeth is mostly determined by the enamel-dentine junction morphology; the enamel-dentine junction plays the role of a primer and conditions the formation of the occlusal enamel reliefs. However, the accretion of the enamel cap yields thickness variations that alter the morphology and the topography of the enamel–dentine junction (i.e., the differential deposition of enamel by the ameloblasts create an external surface that does not necessarily perfectly parallel the enamel–dentine junction). This self-reliant influence of the enamel on tooth morphology is poorly understood and still under-investigated. Studies considering the relationship between enamel and dentine morphologies are rare, and none of them tackled this relationship in a quantitative way. Major limitations arose from: (1) the difficulties to characterize the tooth morphology in its comprehensive tridimensional aspect and (2) practical issues in relating enamel and enamel–dentine junction quantitative traits. We present new aspects of form representation based exclusively on 3D analytical tools and procedures. Our method is applied to a set of 21 unworn upper second molars belonging to eight extant anthropoid genera. Using geometrical analysis of polygonal meshes representatives of the tooth form, we propose a 3D dataset that constitutes a detailed characterization of the enamel and of the enamel–dentine junction morphologies. Also, for the first time, to our knowledge, we intend to establish a quantitative method for comparing enamel and enamel–dentine junction surfaces descriptors (elevation, inclination, orientation, etc.). New indices that allow characterizing the occlusal morphology are proposed and discussed. In this note, we present technical aspects of our method with the example of anthropoid molars. First results show notable individual variations and taxonomic heterogeneities for the selected topographic parameters and for the pattern and strength of association between enamel–dentine junction and enamel, the enamel cap altering in different ways the “transcription” of the enamel–dentine junction morphology.

## Introduction

Tooth is a central element in many fields of investigation especially in anthropology and paleoanthropology as a key tissue for taxonomic identification, phylogenetic or biological inferences (e.g. [1]–[16]). In this regard, many aspects of the teeth size and shape have been considered by researchers over the last century, from detailed description of the occlusal morphology to the characterization of the underlying developmental processes of the tooth form (e.g. [5], [17]) including quantification of various dimensional aspects of the teeth by a wide variety of descriptors (e.g. [3], [12], [18]–[26]).

The recent technological advances in capturing objects' tridimensional morphology by mean of noninvasive methods (e.g. CT and µCT-scans, laser-scanning, …) and the improvement of image analysis tools allowed renewing tooth studies (see e.g. [10], [12], [15], [17], [18], [20]–[21], [24], [26]–[28]). Not only researchers are able to consider an extensive set of dental features in their analyses including crown enamel and enamel-dentine junction, roots and pulp canals (see e.g. [29]–[32]), but also they are able to consider these tissues in their comprehensive tridimensional aspects.

Thus, 3D image analytical tools offer the opportunity to appraise and characterize both morphology and properties of the dental tissues in a quantitative way. On one side geometric morphometric-based methods consist in describing shape by the use of landmarks, semi-landmarks and outline coordinates. These powerful methods allow independently analyzing size and shape and have been successfully applied to tooth shape studies (e.g. [9], [19], [33]–[34]). Such methods describe effectively occlusal outline of the teeth as well as occlusal cusp pattern, i.e. the way the cusp are positioned relatively to each other. On the other side biomechanics like finite element analysis try to explain the functional importance of morphological traits, apart from a metric quantification of the morphological features (e.g. [35]). Between these two poles of the tooth analysis, numerous methods can be found taking into account both morphology and function. Hence, relationships between dental morphology and function may be approached using quantitative descriptors of the tooth surface like microwear analyses and tooth tribology for assessing diet, or Occlusal Fingerprint Analysis for evaluating cusp interlocking pattern and occlusal tooth wear (e.g. [3], [36]–[41]). Besides, tooth form and function can be accessed through topographical analysis of the occlusal surface using GIS software (e.g. [15], [36], [42]). Also, other methods like surface deviation analysis or virtual cartography and morphometric map procedures are routinely applied in medical and anthropological sciences although mostly focusing on thickness measurement of cortical bone and tooth enamel [24], [28], [43]–[46]. Rigorously, assessing dental evolution requires taking into account all the results issued from the above mentioned methods as they provide complementary evidences. In this paper we will focus on data retrievable from topographical analyses. We present an original approach combining 3D topographical analysis of the occlusal enamel surface (OES) and the occlusal enamel-dentine junction surface (EDJ), and their geometrical relationship using surface deviation analysis-based method. We aim to propose a comprehensive characterization of the tooth occlusal morphology using 3D positioning and distribution of topographical (elevation, inclination, orientation) and material (enamel thickness) descriptors of the dental features.

While the use of topographical and surface deviation analysis procedures has been proved to be of significant interest in paleontological and paleoanthropological studies, notably for qualitative and quantitative characterization of the dental form [36], [47]–[51], their potential are still underexploited. Classically, GIS studies recurrently invoke, at various steps, data reduction procedures from 3D to 2D. While such a loss of information in a 2D approach may be rewarded by a better readability of the results and a stronger statistical power, GIS studies tend to dispose of potentially crucial information or to mask numerous features (e.g. re-entrant angles and vertical surfaces linked to particular enamel morphologies, regions under bent cusp tips, protuberances, walls of grooves, regions close to the cervix, or below the maximum crown diameter, are not considered (e.g. [36], [47]–[48]). We present here new aspects of tooth form representation based exclusively on 3D image analytical tools and procedures which allow avoiding such limitations, topographical variables being implemented in a fully tridimensional context.

Moreover, studies considering the relationship between enamel and dentine morphologies are rare, and none of them tackled this relationship in a quantitative way. Yet, the tooth morphology, i.e., the occlusal morphology of the crown enamel develops from the enamel-dentine junction (EDJ, [5], [17], [52]–[53]). The EDJ plays the role of a primer and conditions the formation of the occlusal enamel reliefs. Nevertheless, the way the EDJ acts on enamel morphology albeit of primary interest in dental studies, remains insufficiently characterized and not quantified. For the first time, to our knowledge, it is possible to comprehensively quantify OES (outer enamel surface) and EDJ (enamel-dentine junction) topographical covariations.

We applied our procedure to a set of anthropoid, unworn to slightly worn, left upper second molars. The resulting 3D dataset constitutes an alternative characterization of the tooth form, relevant for dental studies. However a detailed systematic/functional analysis, applied to explicit hypotheses, is beyond the scope of this article and will require a more extensive sample to be achieved in further studies.

## Data Acquisition and Analyses

While a wide variety of noninvasive solutions are accessible for capturing object's tridimensional forms, microtomographic systems (µ-CT) remain a powerful and efficient tool in retrieving detailed aspects of dental components morphology (e.g. enamel occlusal surface, enamel-dentine junction surface) (e.g. [9]–[10], [12], [29], [54]). Our molars sample consisting in two gibbons (*Hylobates sp*), four gorillas (*Gorilla gorilla*), three chimpanzees (*Pan troglodytes*), five modern humans (*Homo sapiens*) and six cercopithecoids (two *Cercocebus sp., two Cercopithecus sp*. and two *Papio sp*.) and one South American monkey (*Lagothrix sp.*). The studied molars belong to the osteological collections of iPHEP/University of Poitiers (19th century) which consist exclusively in bone material (for research and education). None of the molars in our sample were collected from live animals or humans, besides no animals were killed specifically for this study. This sample offers an adequate taxonomic and morphological diversity for appraising our protocols even though an extended sample size is required for testing explicit functional/systematical hypotheses. Molars were scanned using a µ-CT VISCOM X8050 (Centre de Microtomographie at the University of Poitiers).

In order to isolate enamel and dentine materials for each molar, the virtual volumes reconstructed from microtomographic images are processed using automatic segmentation tools with manual correction using ©Avizo v7 commercial software. A review of concepts and practices in 3D image segmentation is beyond the scope of this notes and the reader may refer to the abundant literature on this topic [55]–[58].

**Figure 1:**
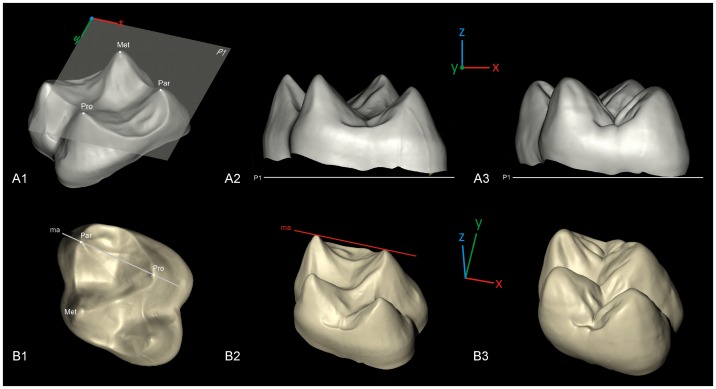
Tridimensional orientation of the molars in the virtual space, example of a*Gorilla*. A1, alignment of the plane defined by the tip of the dentine horns at protocone (Pro), paracone (Par), metacone (Met) to the *x*, *y* plane (P1) of the virtual space; A2, aligned enamel-dentine junction surface; A3, aligned enamel surface. The lowermost point of each molar cervix is set to *(x, y, 0)* so that crown height is measured on a z-positive scale. The *z* axis is positively oriented from the cervix to the occlusal relief of the tooth. B1, the mesial axis (ma), a line joining the tip of the dentine horns at paracone and protocone is set parallel to the *x-axis* of the virtual space; B2, oriented enamel-dentine junction surface; B3, oriented enamel surface. Not to scale.

Once segmentation is complete, the crown enamel is isolated from dentine material and pulps canals. The crown enamel volume data is converted into a polygonal surface (a 3D triangular mesh) corresponding to a set of tridimensional points (nodes) connected by edges. This operation allows partitioning the crown enamel in its inner (enamel dentine junction, EDJ) and outer (outer enamel surface, OES) components (Figure 1). For analyses purposes and in order to minimize the computational load for this note, each EDJ and OES was set to an equivalent reduced amount of polygons by decimation. The decimation procedure was done by a re-tessellation of the original polyhedral surface with tooth-size standardization of the polygonal unit area (i.e., each surface is constituted by polygons of equivalent area, which area depends of the tooth size). Following this procedure, all occlusal enamel and enamel-dentine junction surfaces are constituted by about 22,000 polygons across our sample. Decimation of the original surfaces does not yield significant alteration of the tooth morphology (see Figure S1).

Each surface node and surface triangle is precisely referenced by Cartesian (x, y, z) coordinates in a 3D virtual space. In order to normalize our result, we choose to orient each molar in their 3D virtual space using a common reference plane and axis. While the designation of reference planes for tooth reorientation is an important step for the following computations, the definition of such planes may vary according to the aim of the study, the tooth under consideration, the wear level, the sampled taxa and the authors (e.g. [59]). Hence, here we choose to align the 3D virtual surfaces to a geometrically constructed plane without any preconception on biological or functional meaning, or on reproducibility in a future larger, more heterogeneous sample (i.e., including more disparate taxa and molar morphologies). The only prerequisite, for convenience purpose, was to have the occlusal surface parallel to the viewer plane (e.g. [59]–[60]). First, each molar is aligned to a (*xy)* plane (reference plane) defined by the tip of the dentine horns at protocone, paracone, metacone (Figure 1A). The *z* axis is positively oriented from the cervix to the occlusal relief of the tooth. Second, each molar is realigned in the reference plane by having its mesial axis, a line joining the tip of the dentine horns at protocone and paracone, parallel to the *x* axis of the 3D virtual space (Figure 1B). Finally, for convenience purpose, the lowermost point of each molar cervix is set to *(x, y, 0)* so that crown height is measured on a z-positive scale.

Having all the molars oriented, morphological and topographical data have been retrieved from EDJ and OES. The surface geometry of the molar morphology consists in a matrix of 3D point coordinates and their connections. Each triangle of the polygonal surfaces is indexed and can be attached to a variety of signifiers (e.g. enamel thickness at location, elevation, orientation, and inclination). Hence, not only individual or average signifier values are retrievable from precise location on the molar but also their complete distributions and variations over the whole tooth or a particular region of interest.

### Enamel thickness quantification

The enamel thickness (δ) is computed as the minimum Euclidean distance corresponding to the distance from each OES node to the EDJ closest triangle [12]–[13], [24], [43], [45], [61]–[62], (see also [28] for additional comments). For analyze purposes, each OES node index and its triangle counterpart index on EDJ is stored. The resultant matrix describes a geometrical relationship between the occlusal surface of the enamel, and the occlusal surface of the enamel-dentine junction. This OES to EDJ minimum-distance relationship is used as a basis for comparing OES and EDJ topographic signifiers in subsequent analyses.

### Enamel and dentine topographical signifiers

A normalized vectors field has been computed from the 3D molar triangular mesh and the matrix of normal vector coordinates at each individual triangle of EDJ and OES has been recorded. The vector coordinates are used as raw data for retrieving topographical information of the tooth form, OES and EDJ being treated independently.

Although numerous descriptors may be generated from OES and EDJ, we selected here four simple topographical parameters that have been shown to be of significant interest in dental studies e.g. in ontogeny, taxonomy or in reconstructing diet habits and masticatory behaviors.

The variables are calculated from OES and EDJ polygon coordinates, normalized vector field and δ values. All the computations are fully automated using a program developed under R [63].

For both OES and EDJ we computed enamel and dentine elevation (respectively γ^e^ and γ^d^), inclination (λ^e^/^d^), orientation (ψ^e^/^d^) and curvature (φ^e^/^d^). These topographic variables have been successfully applied, albeit in a different form, to functional, taxonomic or ontogenetic studies in carnivores, multituberculates, rodents and primates (e.g. [12], [47]–[48], [50]; see also [64]–[66].

### Computations

The R program first performs a polygonal indexation then computes the polygon areas (υ), the polygon geometric center coordinates (i.e., its *x*, *y*, *z* position in the 3D virtual space; χ) and the topographical values (γ, λ, ψ). For OES and EDJ, each polygon is referenced by its constitutive edges and nodes and is numbered. This step allows for all subsequent computed values to be precisely associated to its corresponding polygon along with a precise tridimensional location and an area. The area values are used as a size proxy (using the number of triangle is unreliable) to assess the relative occurrence of following topographical variables (e.g. orientation, inclination).

The orientation, the inclination, and the elevation are computed for each polygon of OES (ψ^e^, λ^e^, γ^e^) and EDJ (ψ^d^, λ^d^, γ^d^).

The orientation is defined as the direction of the polygon normal vector in the *xy* plane of the 3D virtual space and is coded on 360°. This parameter is equivalent to the one defined in [22], [47] (see also [48]) for topographical analyses of carnivore, primate and rodent teeth, as a basis for assessing tooth complexity.

The inclination is defined as the angle between the polygon tangent vector in the -z direction (in the plane perpendicular to *xy* and carrying the polygon normal vector) and the horizontal plane *xy*. This parameter thus differs from previously defined *slope* [36] as “the average change in elevation across the surface” ([36] p. 155, see also [15], [48], [50], [65], [67]–[68]). The inclination (λ) is coded on 180°, from 180° for horizontal polygons to 90° for vertical polygons inclination values below 90° describe re-entrant angles (surfaces).

The elevation is computed as the *z* coordinate of each polygon barycenter (χ^z^). This simple parameter allows assessing precisely, for instance, the height of structures of interest (e.g. cusps, dentine horns) relative to various planes (e.g. central basin, cervix) or the relative cuspal heights (e.g. hypocone versus trigonal cusps). Besides, using polygonal *x*, *y*, *z* coordinates allows precisely isolating individual cusp morphologies (i.e., individual cusps being defined by an *x*, *y* interval and a *z* range).

A fourth variable, the mean curvature (φ), has been computed as the mean value of the two principal curvatures values (i.e., the minimum and maximum normal curvatures) for each polygon [69]–[70]. These quantities measure the deviation from flatness of the tooth surface (i.e., the occurrence and magnitude of bulging and hollowing), and are computed for OES and EDJ using ©Avizo v7. In this sense, φ approximates the variable *angularity* (i.e., the second derivative of the elevation; [50]) defined by Ungar and colleagues as the “change in slope across a surface” ([71], p. 3875).

Mean curvatures vary from negative values in strictly concave regions to positive values in strictly convex regions. Also, the levels of φ values provide a quantitative assessment of the convexity/concavity profile; slightly concave or smooth convex regions (e.g. basin, rounded cusp) have low φ values while highly concave or sharp convex regions (e.g. groove, dentine horn apex, crest) show φ values with the highest levels. Regions where a positive and negative principal curvature cancels each other (i.e., flat regions) present φ values equal or close to zero.

**Figure 2:**
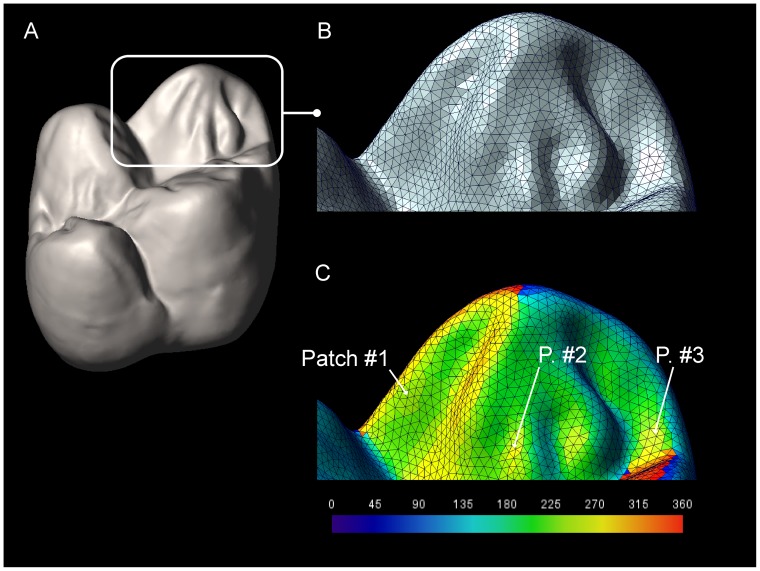
Patch identification for complexity assessment. A, 3D view of a *Gorilla* second upper molar; B, detail of the paracone showing the surface polygonal grid; C, orientation of each polygonal element is coded using a chromatic scale, the contiguous triangles with comparable orientation range form a patch. *Patch#1*, *P. #2* and *P. #3* are three different patches of polygonal elements. Not to scale.

Second, the R program measures the distribution of orientation for OES and EDJ polygons and produces subset of polygons according to a given orientation ranges. For the present study, the orientation intervals are set to 45° producing eight orientation groups (e.g. [47]–[48]). Contiguous polygons within each subset are identified as a patch (Figure 2) and the *complexity* (i.e., the number of these patches within an orientation interval or for the complete orientation range) is computed [22], [47], [49], [72]. Only patch including more than three polygons are considered in this study. For analyses purposes, each patch is associated to its area value as the sum of the areas of its polygon parts.

Finally, the program aims to relate OES and EDJ variables in order to evaluate their correlation. There are no true or sufficient homologous points on either surface to perform such evaluation, and polygonal elements cannot be considered as homologous *per se*. Geometrical properties of the surfaces can be used as an alternative for identifying correspondences between OES and EDJ polygons. Thus, enamel and dentine polygonal correspondence matrix computed for enamel thickness has been used to relate OES and EDJ topographical and morphological polygonal values. The ‘minimum distance’ approach is classically implemented as a 3D surface registrations and comparisons method. Alternative approach such as ‘minimum normal distance method’ generates equivalent correspondence matrix for our surfaces which are defined by a high density of points (i.e. [28]).

The R program yields a matrix in which each polygon of OES, with its morphological and topographical values, is associated to its corresponding EDJ polygon and respective morphological and topographical values.

### Additional indices

The computed raw values (γ, φ, λ, ψ), were used to calculate a set of additional indices as an illustration of potential applications of our 3D method of quantification. Besides, these quantities allow summarizing the observed variations and facilitating comparisons with previous studies.

Two measures of enamel thickness are given: (1) the *average occlusal enamel thickness* corresponds to the δ-mean value for the occlusal region; (2) the *standard occlusal enamel minimum thickness* (δ^st^) as the sum of each polygonal enamel thickness (δ) multiplied by its surface (υ), and divided by the EDJ occlusal tridimensional area (see also [12]–[13], [24], [43], [61], [73]). The marked reliefs such as cusp tips, crests or grooves tend to be described by more polygons than planar surfaces in tridimensional models. This effect is minimized in our sample due to the re-triangulation of the original polyhedral surfaces. However, the enamel thickness values are weighted by their associated polygonal area in order to control for the irregularity of the polygonal mesh.

The relief is an important feature, albeit difficult to characterize, of the crown form. While the relief is, *sensu stricto*, merely the difference in elevation between two sets of points, we define here relief as the combination of three components which are elevation, inclination and area. A marked relief has a maximal elevation for a minimal base area, the average inclination value is high. It is the opposite for a slight relief. Thus, in this study (but see [36], [50], [74]–[75] for an alternative computation), the relief is assessed by the *occlusal relief index* (Γ) which is computed in dividing the product of the area and elevation of polygon which have an inclination higher than 135° by the area and elevation of polygons which have an inclination lower than 135° (most of the polygons of the occlusal surface have inclination comprised in 90–180°).

The inclination variation (λ^v^), which represents the distribution of flat to steep portions of the tooth, is illustrated, for OES and EDJ occlusal regions, as the distribution of the relative areas representing particular inclination intervals. In this respect, inclination range is partitioned in eighteen intervals of 15° each, and corresponding polygonal area is computed within each interval.

In the same manner, three dimensional quantification of the molar occlusal *complexity* is achieved. The orientation range (ψ) being divided in eight intervals of 45° each, the corresponding polygonal area has been computed within each interval for OES and EDJ. The orientation variation (ψ^v^) is illustrated as the distribution of the area representing particular orientation intervals. Besides, the number of patch (ψ^c^), and their associate respective area, is computed for each interval and for the whole orientation range. (see [47], [49], [72], [75]–[76] for various applications).

Although Γ is a meaningful parameter for molar occlusal relief appraisal, features like grooves, basins and fovea, or ridges and crests are best described using mean curvature variable (φ). For instance, φ allows retrieving, for each tooth, the proportion of area consisting in “grooves” (deep and narrow concavity) and “crests” (sharp or pointing convexity). Only data related to “crest” will be presented here. In order to control for the size effect when comparing curvature values between diverse anthropoid molars, curvature is standardized. In this study, we simply adjusted φ values by a factor consisting in ratio of (1) the cubic root of the volume of respectively OES and EDJ of the molar of interest and (2) the cubic root of the volume of respectively OES and EDJ of a reference molar. Standardized mean curvature values are used to compute a *maximum convexity index* (φ^mci^) as the sum of the products of the area of convex polygons by their curvature value, divided by the OES (or EDJ) tridimensional area. For the occlusal surface, this index assesses the convexity level (i.e., sharp features with high φ values are emphasized) along with the convexity expression (i.e., its area representation). These two quantities may appear to be contradictory since sharp features tends to be represented by smaller surfaces. However, this effect is, at least partially, compensated by the fact that sharp features (e.g. crests) tend also to be more elongated than blunt ones.

Although, we did not consider “crest” length in our study, the *maximum convexity index* follows Kay [64], and Anthony and Kay [77] in evaluating the shearing capacity of the molar.

Finally, relationships between OES and EDJ morphological and topographical values is illustrated using two selected variables: (1) the *relative complexity* (ψ^c–e/d^) as the ratio of the OES and EDJ complexity (see e.g. [72]); (2) The *blunting index* (φ^bi^) which measures, for convex features, the degree of which enamel deposit modifies the enamel-dentine junction morphology, that is, for instance, how sharp/pointing morphologies of the dentine are modified in blunt/rounded morphologies by the enamel. The *blunting index* is calculated as the ratio of the EDJ crest area and the EDJ 3D area (φ^p/EDJ^) divided by the ratio of the OES crest area and the OES 3D area (φ^p/OES^).

The morphological and topographical variables have been computed for each tooth of our sample, and a subsample has been used to test for correlation between OES and EDJ. For this present note, our analysis is restricted to the occlusal regions of OES and EDJ. The OES and EDJ occlusal regions have been defined discretely as the regions above a plane parallel to the reference plane and passing respectively by the lowermost point of 1) the occlusal enamel basin for OES, and 2) the enamel-dentin junction basin for EDJ. Other aspects of the tooth morphology and topography, with extension of the ROI to the whole tooth, will be investigated in further studies.

For visualization purpose, all the computed topographical values are respectively mapped onto OES and EDJ using chromatic color scales and presented for the whole tooth.

## Results and Discussion

**Figure 3:**
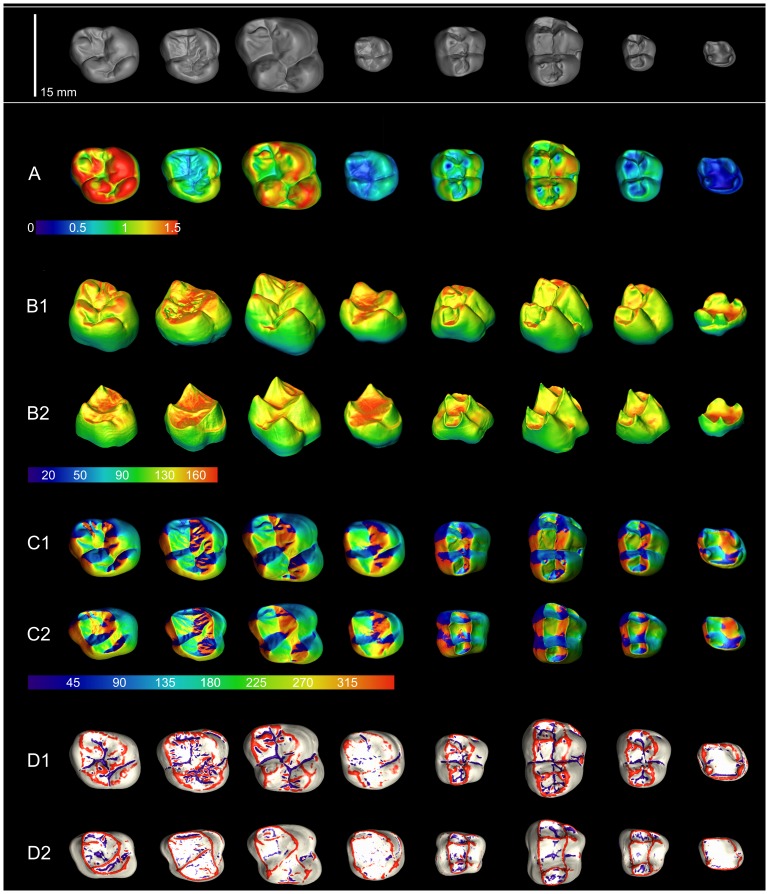
Tridimensional morphometric maps and associated chromatic color scales of the principal topographical parameters for OES and EDJ. A, enamel thickness (mm); B, inclination (degree): enamel (B1), enamel-dentine junction (B2); C, orientation (degree): enamel (C1), enamel-dentine junction (C2); D, standardized curvature: enamel (D1), enamel-dentine junction (D2). The figured molars correspond to a subsample of eight individuals representative of the eight studied genera (from left to right): *Homo*, *Pan*, *Gorilla*, *Hylobates*, *Cercocebus*, *Papio*, *Cercopithecus*, *Lagothrix*. In order to improve the readability, the sizes of the molar representations have been modified in A to D; refer to the grey scale molars (first row) for relative and absolute size.

Computed morphological and topographical maps are reported, for a subsample of anthropoid molars (OES and EDJ) on Figure 3. The topographical variables are reported in Table 1. The results presented in this section aim to provide an example of the potential treatments of the data. Formal testing of taxonomic, developmental or ecological hypotheses is beyond the scope of this note and will be considered elsewhere using extended sample size.

**Table 1:** Measurements and indices for the occlusal surface of enamel and enamel-dentine junction in anthropoid molars.

	Id^1^	δ	δ_sd_	δ^st^	Γ		ψ^c^		φ^bi^	φ^p^		φ^mci^	
***Cercocebus***	#Cb2	0.68	0.17	1.13	0.46	0.42	74	58	1.17	0.32	0.38	0.56	0.89
	#Cb4	0.83	0.16	1.31	0.54	0.48	63	72	1.14	0.32	0.37	0.57	1.05
***Papio***	#Pp3	1.22	0.16	2.03	0.28	0.33	80	82	1.16	0.30	0.34	0.54	0.92
	#Pp4	1.09	0.18	1.65	0.29	0.25	84	72	1.09	0.30	0.32	0.55	0.87
***Cercopithecus***	#Cc1	0.51	0.12	0.71	0.34	0.25	65	59	1.22	0.30	0.36	0.55	0.89
	#Cc2	0.63	0.13	0.95	0.34	0.28	67	58	1.21	0.29	0.35	0.55	0.90
***Gorilla***	#Go1	1.15	0.18	1.58	0.37	0.47	79	53	1.01	0.29	0.29	0.47	0.54
	#Go2	1.09	0.23	1.56	0.50	0.76	88	84	0.94	0.30	0.28	0.49	0.58
	#Go3	0.92	0.14	1.18	0.64	0.68	74	78	1.01	0.31	0.31	0.54	0.66
	#Go4	1.01	0.16	1.29	0.62	0.69	76	68	0.98	0.31	0.31	0.55	0.65
***Homo***	#Ho1	1.44	0.21	2.12	0.66	0.56	60	78	1.02	0.26	0.26	0.41	0.63
	#Ho2	1.41	0.22	2.11	0.81	1.17	82	87	0.82	0.35	0.29	0.55	0.63
	#Ho3	1.50	0.24	2.42	0.89	0.63	67	60	1.09	0.23	0.24	0.33	0.60
	#Ho4	1.48	0.22	2.09	1.12	0.57	60	59	1.12	0.22	0.25	0.34	0.62
	#Ho5	1.44	0.31	2.44	0.97	0.72	74	83	1.10	0.29	0.31	0.47	0.66
***Pan***	#Pa1	0.83	0.17	1.30	1.22	1.43	86	82	1.02	0.29	0.30	0.47	0.67
	#Pa2	0.82	0.22	1.25	1.11	1.39	127	94	1.04	0.27	0.28	0.45	0.62
	#Pa3	0.91	0.16	1.37	1.35	1.54	89	90	0.93	0.32	0.30	0.53	0.76
***Hylobates***	#Hy1	0.54	0.10	0.83	0.90	1.24	47	59	0.98	0.28	0.27	0.40	0.58
	#Hy2	0.53	0.12	0.78	1.06	1.35	51	63	1.01	0.27	0.27	0.39	0.57
													
***Lagothrix***	#Lx1	0.30	0.10	0.42	0.56	0.91	60	64	0.87	0.29	0.25	0.51	0.66

1. Id, specimen number; δ/δ_sd_, mean/standard deviation of occlusal enamel thickness; δ^st^ standard occlusal minimum enamel thickness; Γ, occlusal relief index of OES (first column) and EDJ (second column); ψ^c^, number of patch for OES (first column) and EDJ (second column); φ^bi^, blunting index; φ^p^, relative crest area for OES (first column) and EDJ; φ^mci^, maximum convexity index for OES (first column) and EDJ.

### Enamel thickness

**Figure 4:**
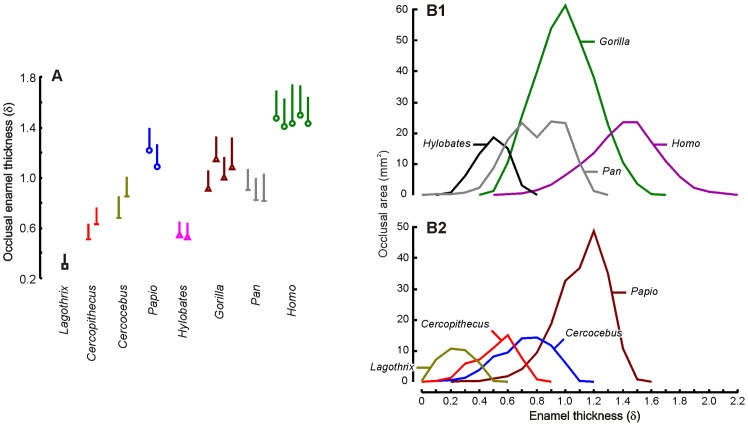
Enamel thickness variation. A, occlusal enamel thickness (δ, whisker diagrams for mean and standard deviation in millimeter); B, enamel thickness distribution (within taxon average, mm) in hominoids (B1) and cercopithecoids/*Lagothrix* (B2) relative to the corresponding area of expression on the molars (mm^2^).

The computed enamel thickness values for the occlusal region of OES (δ) are reported on Figures 3A and 4 (mean and standard deviation are listed in Table 1). Our data are in accordance with previously reported thicknesses, albeit using different variables (e.g. AET *average enamel thickness* [24], [43], [46], [73]; see also AMT *average minimum thickness* [61]). *Homo* presents the greatest average enamel thickness within hominoids then *Gorilla* and *Pan* while highest values are recorded in *Papio* within cercopithecoids (Table 1, Figure 4A). Such hierarchy is maintained using enamel thickness adjusted for molar size (δ^st^) though lowest *Gorilla* values being more similar to those of chimpanzees. The tridimensional quantification of enamel thickness allows examining its variation over the occlusal region of the anthropoid molars. The Figure 4B illustrates the distribution of the occlusal enamel thickness according to its area of expression within each taxon (see also Figure 3A). The enamel thickness variance is highest in *Homo* and closely followed by *Gorilla*/*Pan* then *Papio*/*Cercocebus*. The modern *Homo sapiens* presents also the highest range of δ values (from 0.5 to 2.2 mm on average) suggesting a more heterogeneous distribution of the enamel over the enamel-dentine junction in this taxa. Within modern humans, 42% of the enamel surface corresponds to a δ higher than 1.4 mainly at the protocone (lingual) and at the hypocone. The gorilla/chimpanzee and the genus *Papio* present comparable ranges of δ values albeit with different distributions. The patterns of enamel thickness distribution show notable disparities between taxa. Within hominoids, *Pan* presents a characteristic “bi-modal” shaped curve with comparable representation of two enamel thickness classes, 0.6–0.7 mm and 0.8–1.0 mm. The first class represents thickness at the large central basin while second class corresponds to thickened area around the crown at the cusp level. The gibbon, the gorilla and the modern human show unimodal distributions of enamel thickness. Within cercopithecoids, *Papio* presents the widest δ range (from 0.2 to 1.6 mm). The three cercopithecoid genera show similar patterns of distribution with thicker enamel at the cusps/lophs level and thinner enamel at central basin and distal/mesial fovea (indicated by a step in the δ distribution; Figure 4B2).

### Orientation

**Figure 5:**
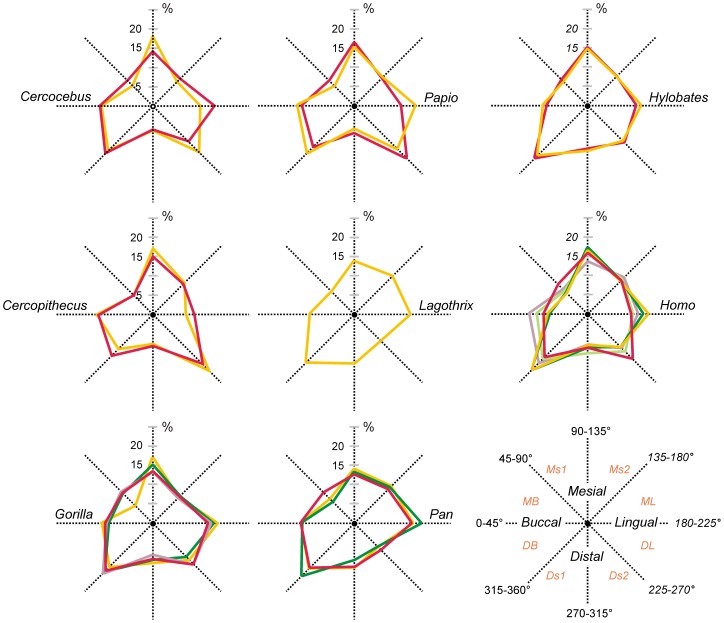
Variations of orientation of enamel occlusal surface. Each profile corresponds to the relative proportion of area expression (in percentage) of the eight orientation intervals. Since orientation is coded on 360°, the relative contribution of the orientation intervals is given in polar coordinates. The schematic drawing (lower right) indicates the direction of orientation for the considered surface according to the tooth orientation. Thus, for instance, polygons for which normal vector is in the 90–135° intervals correspond to a mesially oriented surface. Ms1, Ms2, mesial quadrants; Ds1, Ds2, distal quadrants; MB, DB, mesio- and distobuccal quadrants; ML, DL, mesio- and distolingual quadrants.

The variation in orientation (ψ^v^) for enamel and enamel-dentine junction occlusal surfaces is illustrated in Figure 3 (Figure 3C1, 3C2) and Figure 5; complexity (ψ^c^) is reported in Table 1.

Considering the respective area of expression of orientation intervals, ψ^v^ evidently first depicts overall molar morphology. Thus, elongated (rectangular) molars tend to have proportionally higher area of expression for lingual and buccal orientation intervals than quadrangular molars (Figure 5). However, this expected pattern is altered by the presence of occlusal features such as cusps, grooves, crests, and lophs. Both shape and development of these structures influence the area expression of orientation intervals. While a rounded cusp tends to add roughly equivalent area in each interval, features like crests, grooves or lophs will act only on particular orientation. Hence, ψ^v^ may present clear interspecific discrepancies (an analyze of variance of orientation profiles using shape coordinates show significant differences between cercopithecoids and hominoids at p<0.05 for OES and at p<0.01 for EDJ), but is expected also to document subtle variation related to disparate within-taxon expression of occlusal features.

Theoretically, each orientation interval is expected to represent 12.5% of the total area. The greater deviations from this pattern are found in cercopithecoids while the hominoids tend to exhibit a more uniform distribution of the orientation intervals (see the different types of orientation profiles in Figure 5).

The orientation intervals which present the highest area proportions correspond, on average, to distal (Ds2) and mesial (Ms2) quadrants in *Cercopithecus and Papio* (includes lingual side of the tooth and lingual portion of the buccal cusps). It is the distobuccal interval (DB) for all the other genera followed by the mesial quadrant (Ms2) in *Cercocebus*, and *Homo* and *Hylobates*; by the distolingual quadrant (DL) in *Lagothrix*, *Gorilla* and *Pan*.

Although the enamel-dentine junction surface (EDJ) does not present, overall, contrasting information with OES for ψ^v^ (see Figure S2), a greater variation is seen for orientation intervals' representation within and between taxon.

Considering *complexity*, our results depart from those previously reported in the literature for anthropoids (Table 1; see e.g. [72]). The observed dissimilarities can rely on different sample composition; the *complexity* presents individual variability and variation is also expected to occur between species within a same genus (e.g. within *Hylobates*, *Cercopithecus*). Besides, distinct methodologies (e.g. we limit our results to the occlusal portion of the molar) could also explain the results inconsistencies, and several aspects are suggested to alter the complexity values. First, it is worth noticing that our study is based on a 3D triangular representation of the molar surface while other studies rely on squared pixels representation of a 2D projection. Such methodological distinction is, alone, prone to lead to perceptible variation in patch count. Besides, the minimum number of contiguous cells/polygons defining a patch varies according to author (usually from 3 to 6 [47], [49], [72], [75]–[76]; 3 in this study). This threshold value directly influences the number of patch, i.e. the complexity, and is contingent of two inter-related factors that account for resolution: the cells/polygons size and the tooth size (the number of cells/polygons). Though procedures of tooth/polygon size normalization have been applied in the literature (see e.g. [72]), the combined effect of the minimum number of cells and the surface resolution in numbering patch remains unclear (see e.g. Figure S3). Besides, the integration of particular features like re-entrant or vertical surfaces in our models may also account for ψ^c^ dissimilarities between the present note and other results. Finally, the tessellation techniques producing the enamel and enamel-dentine junction surfaces may generate various kinds of noise (i.e., incorrect position or orientation of a set of polygons). Although various techniques exist for noise reduction, the alteration of the original surface as well as remnant noise may also account for differences in *complexity* values between studies.

All the herein aspects are prone to modify ψ^c^ values between and within taxa. It suggests, at least, that a detailed examination of the factors affecting the patch number is needed for assessing the relevance of *complexity* in later studies. Indeed, the interpretation of this quantity turns out to be questionable when one considers the respective contribution of each orientation interval to the total value. Thus, substantial variations occur between taxa as expected, but also within taxa (see Figure S4). The contribution of each orientation interval to the total *complexity* notably differs between individuals with a maximum number of patches being represented by distinct orientations according to specimens. For instance in *Homo*, two individuals shows a greater ψ^c^ value for the orientation 270–315°, one for 0–45°, one for 180–225° and one for both the intervals 45–90° and 135–180°. Hence, if one considers that the total *complexity* has significance, the way this quantity is acquired is highly variable. It suggests that the total *complexity* aggregates patches with randomly generated orientations over the occlusal molar surface. In this respect, it seems necessary to reconsider *complexity* in taking into account, for instance, the angulation between contiguous patches. Indeed, a similar *complexity* (i.e., an equivalent number of patch) may reflect different morphologies, contiguous patches yielding sharp edges or smooth transitions may have different functional meanings.

### Inclination

**Figure 6:**
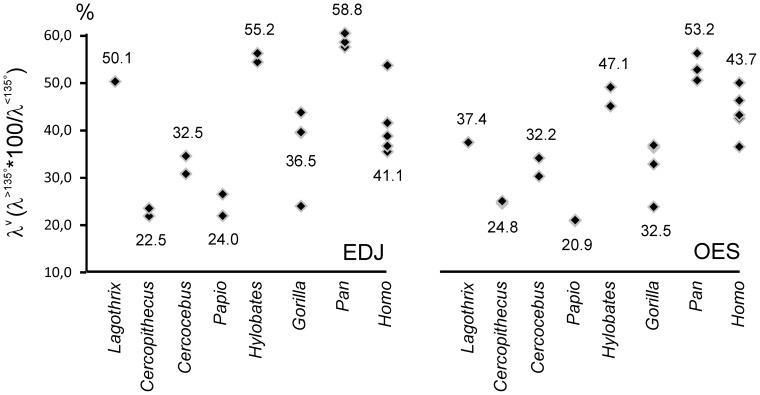
Variation of occlusal enamel and enamel dentine-junction inclination (λ^v^). Values for λ^v^ are reported for each specimen, for both EDJ and OES. λ^v^ is computed as the area of expression of polygons with inclination higher than 135° relative to the area of expression of polygon with inclination lower than 135°. The average λ^v^ is given for each taxon.

**Figure 7:**
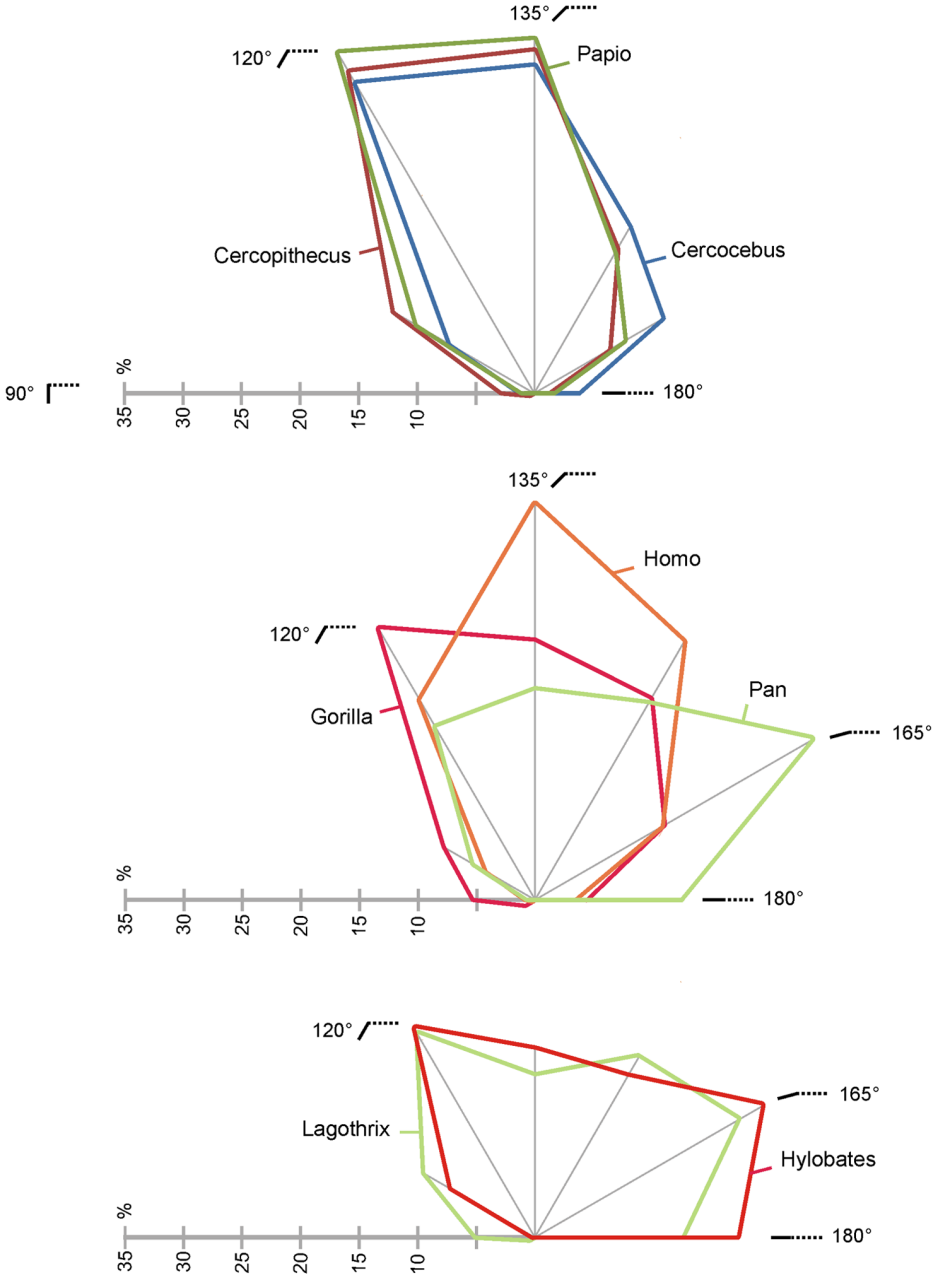
Inclination profiles of anthropoids molars. The profile corresponds, for each taxon (average data), to the EDJ relative proportion of area of expression of inclination intervals (increment is 15°).

The variation in inclination (λ^v^) for enamel and enamel-dentine junction occlusal surfaces is illustrated in Figure 3 (Figure 3B1, 3B2) and Figure 6–7. In this study, the inclination is reported as the variation of the individual polygon values for occlusal OES and EDJ, measures of average inclination were not considered here.

Using the relative area of expression of inclination intervals for describing λ^v^ (Figure 6), the enamel-dentine junction surface is rather flat in Chimpanzee, *Hylobates* and *Lagothrix*. Contrarily, the area corresponding to steep EDJ is relatively more expanded in cercopithecoids, *Gorilla* and modern humans. The enamel cap yield noticeable modification of the EDJ pattern with a relative flattening of most of the molars (Figure 6). Indeed, the ratio of λ^v^ proportions of EDJ and OES is above 1.0 in *Lagothrix* (1.34), *Hylobates*, *Papio* (1.15), *Gorilla* and *Pan* (1.10). It is close or slightly below 1.0 in *Cercocebus* (1.0), *Homo* (0.94) and *Cercopithecus* (0.91). Of course, such result needs to be confirmed using a greater sample; it also needs to be detailed with a clear distinction of the influence of dental traits such as crests, grooves and cusps in inclination values. Further studies will take into account the influence of the amount of enamel deposition (enamel thickness) in the modification of the flat/steep relative areas (as presented in paragraph “covariation” here after).

The relative contribution of particular inclination interval to the tooth surface is illustrated in Figure 7. For simplification and to avoid lengthening this technical note, we choose to present results for EDJ alone, with emphasis on interspecific variation rather than intraspecific variability (inclination profiles for OES are illustrated in Figure S5). The cercopithecoids exhibits similar inclination profiles, distinct from other anthropoids in having higher area expression for rather steep surfaces (from 105° to 135°). Accordingly, an analyze of variance of inclination profiles using shape coordinates show significant differences between cercopithecoids and hominoids at p<0.01 for EDJ and p<0.05 for OES. In *Gorilla*, the interval 105°–120° is also well represented but this taxon tends to show more evenly distributed areas in term of inclination. The modern *Homo sapiens* profile is comparable to those of cercopithecoids albeit with a shift towards horizontal surface, i.e., represented by the 120°–150° inclination intervals. Besides, the genera *Pan*, *Hylobates* and *Lagothrix* share an important area expression of the inclination interval 150–165°. However, while the second greatest area expression corresponds also to rather horizontal surfaces in *Pan* (135°–150°), it corresponds to rather steep surfaces in *Hylobates* and *Lagothrix* (105°–120°).

### Occlusal relief index

The computation of the *occlusal relief index* (Γ) is listed in Table 1 for OES and EDJ (see also Figure S6). The chimpanzee presents the highest *occlusal relief index*, indicating a relatively horizontal (i.e., flat), occlusal surface with rather low, smooth reliefs. The computed Γ values are on average higher for EDJ than for OES (although not significantly in our sample; t = −0.44, p = 0.66, df = 40). It suggests that enamel deposit slightly modifies the EDJ in accentuating reliefs. Such accentuation is also observed in *Hylobates*, *Lagothrix*, and one *Homo* specimen, and to *Gorilla* to a lesser extent. No significant relationship exists between “relief accentuation” (i.e., the ratio of Γ^OES^ and Γ^EDJ^) and *average occlusal enamel thickness* (r = 0.41, p>.05; the relationship is significant when “relief accentuation” is compared to a scale-free average occlusal enamel thickness as the quotient of average occlusal enamel thickness and occlusal dentine volume [24]; r = 0.59, p<0.01). However, since the enamel is not evenly deposited over the EDJ, local variation of the enamel thickness certainly accounts for the observed differences between Γ^EDJ^ and Γ^OES^. The *occlusal relief index* is the lowest in cercopithecoids (outside of one *Gorilla*), with comparable values for EDJ and OES. This result, indicating molars with marked relief, is in accordance with the typical observed morphology in these genera. The modern humans exhibit notable variation for Γ^EDJ^ and Γ^OES^, the Γ values being intermediate between those computed in cercopithecoids and chimpanzees. Also, except for one individual, the *occlusal relief index* is lower for EDJ than OES. It suggests, contrary to other hominoids, that the enamel-dentine junction surface is smoothed out by the enamel deposit. As noted previously our *occlusal relief index* (Γ) intrinsically differs from classical relief index reported in the literature (see e.g. [36], [42], [50], [68], [74]), but the meanings of these indices remain unclear. While a significant correlation exists between Γ and *relief index* (i.e., the ratio of the surface area of the enamel crown, and the surface area of the crown's projection into an occlusal plane; r = −0.91 at p<0.01), the two indices vary conversely (see Figure S6). Although Γ appears to be more sensitive to relief irregularity and presents higher variance than *relief index* for our sample (see Figure S6), it is clear that similar index values may arose from different molar morphologies (and *vice versa*). It suggests, at least, that improvement of Γ (and/or *relief index*) is needed before use in ecological (e.g. diet) or morphological studies. For instance, a better appraisal of proportion of negative *versus* positive reliefs (e.g. groove versus crests) or a better distinction between primary (e.g. cusp, loph) versus secondary reliefs (e.g. cusp crests, crenulations, grooves) is required.

### Curvature

**Figure 8:**
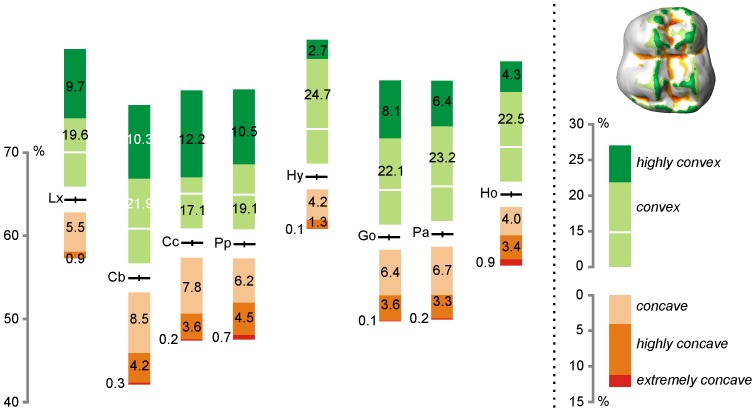
Standardized mean curvature values of enamel occlusal surface. The position of each taxon on the graph corresponds to the area proportion of enamel occlusal non-curved surface (left axe, horizontal line is for within taxon average value). The bars illustrate the associate proportion of area of expression of convex (light green)/concave (light orange) to highly convex (green) and extremely concave (red) surfaces (average value, see right panel).

The variation of curvature (φ^v^) for enamel and enamel-dentine junction occlusal surfaces is illustrated in Figure 3 (Figure 3D1, 3D2) and curvature indices (φ^p^, φ^bi^, φ^mci^) are reported in Table 1. The Figure 8 presents the area proportion of standardized curvature values for OES in our sample (φ values for EDJ are illustrated in Figure S7). The area proportion of weakly concave/convex surface (*wcs*) is high for the selected anthropoid molars and varies from 54.9% in *Cercocebus* to 67.1% in *Hylobates* (Figure 8). This value is lower in cercopithecoids than in hominoids (t = −2.26, df = 18 at p<0.05) illustrating a somewhat less unrelieved “landscape” in monkeys. Besides, salient (convex to highly convex) reliefs are relatively more extended in cercopithecoids with a greater area for highly convex surfaces, indicator of the shearing capacity of these molars. Highly convex areas correspond to the apex of the cusps, to the crests, lophs and to the mesial and distal marginal ridges. At comparable *wcs* values, *Gorilla* and *Pan* presents a relatively lower proportion of highly convex surface than *Cercopithecus* or *Papio* (t = 4.96, df = 11 at p<0.01). Convex surfaces are relatively more extended in the African Apes while concave to highly concave surfaces are similarly developed. The relative reduction of highly convex surface is accentuated in modern humans, a taxon which presents the second highest relative area for *wcs*. In *Homo*, the proportion of convex surface remains high (22.5%, Figure 8) as well as the area proportion of highly to extremely concave surfaces (including grooves).

**Figure 9:**
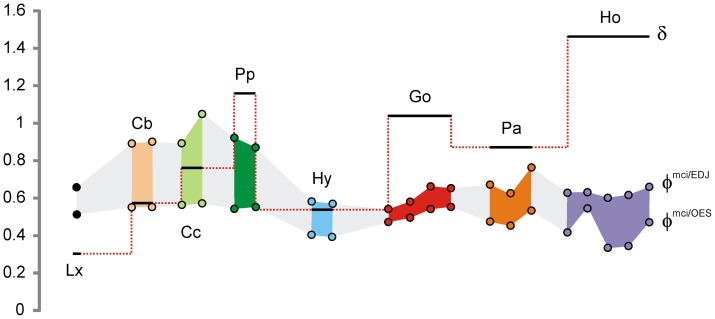
Maximum convexity index for enamel and enamel-dentine junction, comparison with occlusal enamel thickness in anthropoids. Each specimen is represented by two circles, one for EDJ (φ^mci/EDJ^) and one for OES (φ^mci/OES^). The average occlusal enamel thickness (mm) is given for each taxon (horizontal line). Lx, *Lagothrix*; Cb, *Cercocebus*; Cc, *Cercopithecus*; Pp, *Papio*; Hy, *Hylobates*; Go, *Gorilla*; Pa, *Pan*; Ho, *Homo*.

The observed curvature pattern of the selected anthropoid molars is corroborated by the *maximum convexity index* (φ^mci^) and the *blunting index* (φ^bi^, Table 1). Besides, theses indices provide important hints on curvature modifications of the enamel-dentine junction by the enamel deposit. The Figure 9 presents the distribution of φ^mci^ for OES and EDJ in our sample. The *maximum convexity index* is, on average, higher in cercopithecoids than in hominoids (t^EDJ^ = 9.84, df = 16, p<0.01; t^OES^ = 2.77, df = 16, p<0.05), the latter exhibiting nevertheless substantial individual variation. Also, the φ^mci^ values for EDJ are consistently higher than their OES equivalent, suggesting that enamel deposit tends to blunt the enamel-dentine junction (see also [9]). It is particularly true for cercopithecoids which present the greater discrepancies for φ^mci^ between EDJ and OES. Such variation, in accordance with the results observed for φ^bi^, does not seem to be related to molar size or to enamel thickness (Figure 9). Further investigation is necessary to substantiate our observation and to interpret the meaning of such a “blunting” effect of the enamel-dentine junction in cercopithecoids.

### Covariation

**Figure 10:**
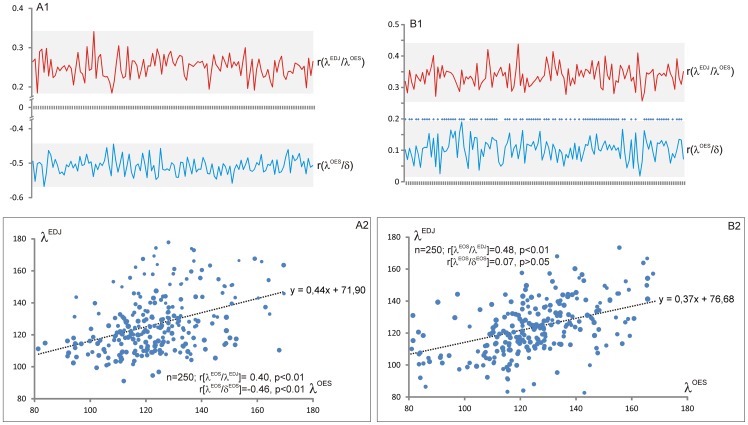
Relationships between inclination of OES, minimum enamel thickness and inclination of EDJ. Example of *Cercocebus* (A) and *Gorilla* (B). The correlations and their significance between λ^OES^ and δ, and between λ^OES^ and λ^EDJ^ have been computed for about 130 subsamples of 1000 inclination and thickness values (resample with replacement on the original data) for each specimen. The correlations are significant for all λ^OES^ and λ^EDJ^ comparisons in *Cercocebus* (A1, red profile) and *Gorilla* (B1, red profile). The OES inclination is significantly correlated to the enamel thickness (albeit negatively) in *Cercocebus* (A1, blue profile) while λ^OES^ and δ present significant positive correlation in 72.1% of the cases in gorillas (B1, blue profile; significant correlations are marked with a blue diamond). A simplified version of the observed relationship is given for *Cercocebus* (A2) and *Gorilla* (B2) from a subsample of 250 inclination and thickness values (resample with replacement on the original data). The surfaces of the dots in A2, B2 represent minimum enamel thickness value (scale *10).

A correlation matrix between nine variables (δ, γ^OES^, λ^OES^, ψ^OES^, φ^OES^, γ^EDJ^, λ^EDJ^, ψ^EDJ^, φ^EDJ^; significance at p<0.01) has been computed for each specimen. Our sample presents significant correlations in most cases with, on average, 78.5% of variable comparisons being significant per specimen. However, only few variable comparisons show moderate to strong correlation (from r = 0.30 to r = 0.73 at p<.01). Highest correlations occur for δ/φ^OES^ (e.g. r = 0.69 in *Lagothrix*; r is about 0.60 in *Gorilla*) and for γ/λ and γ/φ comparisons for both OES and EDJ (e.g. r(γ^EDJ^/λ^EDJ^) = 0.73 in *Hylobates*; r(γ^OES^/φ^OES^) = 0.48 to 0.50 in *Cercopithecus*). Considering the relationship between the enamel surface and the enamel-dentine junction surface, a significant correlation is repetitively observed for γ^OES^/γ^EDJ^, γ^OES^/φ^EDJ^, and λ^OES^/λ^EDJ^. For this present note we will focus on the λ^OES^/λ^EDJ^, and λ^OES^/δ relationships with the example of one *Cercocebus* specimen and one *Gorilla* individual. The Figure 10 presents the correlations, and their significance, of λ^OES^ and δ, and of λ^OES^ and λ^EDJ^ comparisons. Regarding the amount of data (about 5000 and 7000 value for each variable), we choose to evaluate the correlation between λ^OES^, λ^EDJ^ and δ, using about 130 subsamples of 1000 inclination and thickness values (resample with replacement on the original data) for each specimen. The correlations are significant for all λ^OES^ and λ^EDJ^ comparisons in *Cercocebus* and *Gorilla*, suggesting that the enamel-dentine junction inclination primarily constrains the enamel inclination. Nevertheless, the coefficient of correlation illustrates a weak to moderate relationship (r varies from 0.18 to 0.43) with significantly lower correlation in *Cercocebus* than in *Gorilla*. This result indicates that λ^EDJ^ cannot account alone for the observed λ^OES^ variation. In this respect, it is reasonable to consider that variation of enamel thickness may alter the relationship between λ^OES^ and λ^EDJ^. The OES inclination is significantly and highly correlated to the enamel thickness (albeit negatively) in *Cercocebus* (Figure 10A) while λ^OES^ and δ present significant positive correlation in only 72.1% of the cases in gorillas (with weak coefficient of correlation not exceeding 0.19; Figure 10B). Hence, the enamel does not act similarly in *Cercocebus* and *Gorilla* in modifying the enamel-dentine junction inclination. Such discrepancy might be explained by different level of enamel thickness, thicker in *Gorilla* than in *Cercocebus*. Also, since the enamel is not evenly distributed over the occlusal EDJ (e.g. thinner in the central basin, laterally thicker on cusps), local variations of the enamel thickness certainly affect the observed relationship between occlusal λ^EDJ^ and λ^OES^. A reanalysis in a more restricted region of interest (e.g. one cusp), over a larger sample, would yield to a better understanding of the relationship between λ^EDJ^ and λ^OES^ and the role of the enamel thickness in the modification of λ^EDJ^.

## Conclusions and Prospects

Our approach is shown to be a useful tool for characterizing tooth form and is expected to provide developments in several fields of dental studies. The topographic parameters originally described by [50] are quantified in three dimensions without concealing the information carried by reentrant angles or vertical walls (e.g. [48], [65]). Such information could be suitable in studying particular aspects of the tooth morphology. For instance, small insectivorous mammals classically have sharp cusps, of which they use the entire surface to penetrate the cuticle of arthropods. But these cusps are often slightly curved, which hides areas and corresponding data (e.g. the orientation patch number) located in the constituted reentrants during a 2D complexity analysis. Similarly, muroid rodents have distributions of inclination values that are assumed to vary considerably according to their occlusal grades [48]. Among them, rodents with extreme tooth morphology such as the Arvicolinae, for instance, display very important reentrants, because of the inclination of the pillars of their hypsodont molars, which may bias the comparison with brachyodont muroids.

Also, though several studies have recently undertaken three-dimensional analyses of the tooth structural morphology (e.g. [9]–[11], [78]), the covaration between enamel and enamel-dentine junction is for the first time, to our knowledge, considered in a quantitative way using their geometric relationship. Besides, the proposed method enables an accurate localization of every features of the occlusal morphology thanks to the collected 3D data. The integration of topographic parameters along with their precise localization provides a new opportunity to study the variation of these parameters (i.e. their distribution) on the whole surface of the tooth, or for more reduced regions of interest (e.g. the occlusal surface in this work, but applies also to particular features like cusps). The size component of these distributions, i.e. their surface extension, completes their study. Targeting the modifications through localization is also an opportunity to define precisely dental cusps and crests, of which definition is sometimes difficult in some species of mammals, especially when it comes to evaluating the intraspecific variation of a taxon or the evolution of a morphotype within an evolutionary lineage. Established parameters can thus be used to determine thresholds for which tooth elements can be considered as cusps or accessory cusps, gutters or crests.

The approach proposed in the present paper raises several important questions that were not specifically addressed in previous studies but may be discussed through examples selected in our dataset: 1) the taxonomical and functional signal carried out by the topographical descriptors; 2) the relationship between dental complexity and diet; 3) the influence of the enamel layer on topographic parameters including curvature.

**Figure 11:**
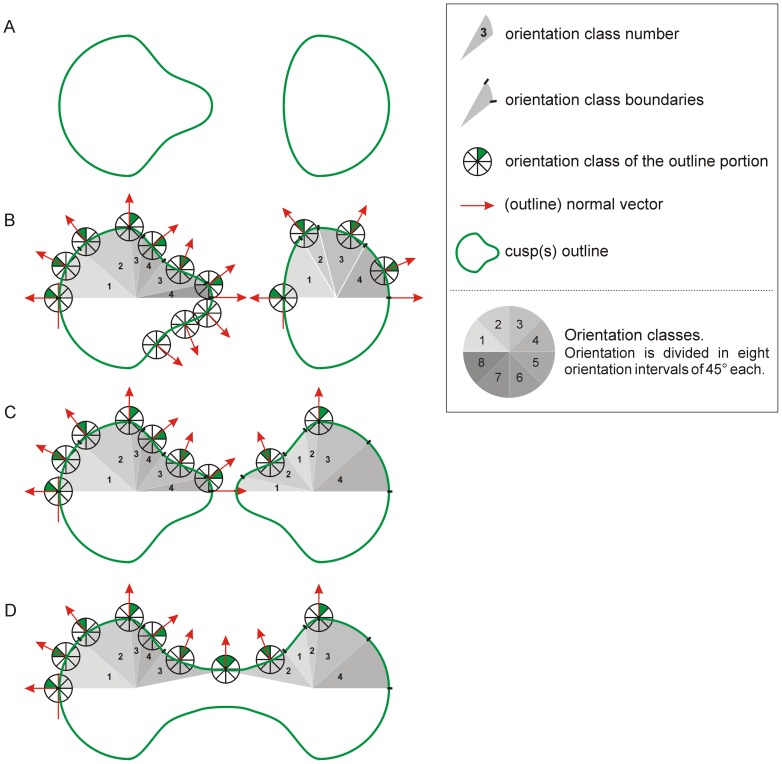
Measuring the bilophodonty by the mean of polygon orientation distributions. Example of a theoretical case of two cusps (outlined in green) showing various degrees of bilophodonty; a & b illustrate distinct lingual and buccal cusps, c development of buccal and lingual crests at lingual and buccal cusps, d, loph joining lingual and buccal cusps. The normal vector (red arrow) describing each point of the cusp outline (or polygons for tridimensional data) allows retrieving orientation values along the cusp contour. The cusp contour can be decomposed in different portions describing same orientation intervals (e.g. 0–45° or 90–135°, only mesial halves of the cusps is shown) numbered from 1 to 8. The distribution and the relative contribution of the orientation intervals to the shape of the cusp allow quantifying the development of bilophodonty. Hence for instance, complete bilophodonty is marked from c to d by the deletion of orientation classes 4 and 1 between cusps on their mesial side.

First, the selected topographic parameters (e.g. orientation, inclination) enable to distinguish high-ranking (family level) and low-ranking (genus level) taxa among anthropoids as evaluated here using unworn to slightly worn molars. This result is expected since topographic parameters obviously describe morphological aspect of the tooth. Thus, bilophodont molars in cercopithecoids exhibit orientation and inclination values in accordance with the development of transverse lophs between cusps contrary to bunodont molars in hominoids. Besides, curvature seems to be a relevant parameter for accounting subtle differences within cercopithecoids and hominoids allowing describing morphological features potentially associated to the tooth function, like sharpness versus bluntness [79]–[80]. Thus, the distinctions emphasized in the present work can complement results from geometric morphometrics in which occlusal cusp pattern or even crown outline are described (e.g. [19], [33]). Our preliminary results highlight the potential of topography as a taxonomical tool which will have to be assessed on variously worn specimens considering that extreme tooth wear may question such a potential (but one can reach the same conclusion whatever the method of investigation used for interpreting morphology in term of taxonomy). Besides, our approach may also enable to quantify the emergence and the retention of diagnostic dental features such as bilophodonty in Old World monkeys (to decipher the respective part of the OES and EDJ in the development of that feature; Figure 11). In this way the evolution of primates is characterized by the recurrent emergence of the hypocone in their different lineages as in Adapidae [81], likely according to distinct evolutionary pathways. The variables featured in this work as well as the localization data should contribute to the study of the various modes of emergence of this key character.

Moreover the spatialized approach developed in this work offers the opportunity to practically assess the ‘local’ influence of some topographic parameters initially calculated for the entire crown, as inclination [48] and complexity (e.g. [22]). More generally, targeting the modifications through localization is a way to appraise, on the occlusal surface, the topographical variations of each element in respect to the functional constraints induced by diet (e.g. [47]) and occlusion [48], [65]. For instance, the measurement of shearing crest development on primate molars is regularly used to infer the diet of fossil species (e.g. [64], [78], [82]). However, while various types of crest reliefs are present on the occlusal surface of the tooth, their location, height, length, orientation, and sharpness may have different functional meanings depending of the body size, dietary habits, and masticatory phases of the taxa under consideration. The 3D localization and distribution of the parameters presented in this work is expected to allow accounting for these patterns of variation when studying particular structures such as dental crests. Similar work could be undertaken to evaluate the functional significance, if any, of the grooves of the occlusal surface.

Second, analyzing the dental topography in its comprehensive tridimensional aspect yields unanticipated results somewhat contrasting with previous studies. For instance, methodological issues may explain the variations observed between our results about topographic complexity and those of the literature (e.g. [72]), but also suggests that previous analyzes did not take into account of the entire tooth complexity. The complexity index defined by [22] illustrates the relationships between the number of patches observed on the occlusal surface and diet, the patches being supposed to reflect the tooth breakage sites. Carnivorous species have a reduced number of patches, while in herbivorous species this number is high. The relationship between complexity and diet seems to be more difficult to interpret in primates (see also [72]): despite our sample did not include purely insectivorous primates like *Tarsius*, the complexity variable should have highlighted differences between omnivorous taxa incorporating insects in their diet (e.g. *Pan*, *Cercocebus*), and more exclusively frugivorous taxa (e.g. *Cercopithecus*, *Hylobates*), which was not the case. In this respect, a sample encompassing more taxa, i.e. having a greater variation in dietary habits, would be more suitable to assess relationships between complexity and diet in this group. If a link between topographic complexity and all diets should be confirmed by 3D analyses, localization should determine whether the adaptive signal is carried exclusively by the total number of patches, or is the byproduct of each orientation class' contributions to the total complexity. Our study suggests that similar values of complexity can be obtained by different relative contribution of orientation ranges in individuals of the same species. Hence equivalent complexity may arise from different occlusal patterns and therefore may have different functional outcomes in the context of similar masticatory movements. On the long range, the reappraisal of similar/different morphologies for comparable diet in tridimensional and integrative (morphology, topography, size) way is necessary.

Finally, the localization of the enamel topographic parameters and their matching with the dentine topographic parameters, associated with localized quantifications of the enamel thickness, is prone to improve our understanding of the respective influences of the enamel-dentine junction and the enamel layer over crown morphology. In the early stages of tooth development, the enamel-dentine junction undergoes folding controlled by cellular signaling centers known as the secondary enamel knots, ultimately responsible for cusp patterning (e.g. [83]). This folding takes place prior to the differentiation of the enamel-forming ameloblasts from the inner enamel epithelium and thus before the secretion of the enamel layer, which partially modifies the morphological signal carried by the enamel-dentine junction. The morphology of the enamel-dentine junction and the thickness variations of the enamel layer appear to be controlled by distinct genes (e.g. [84]–[85]). Our preliminary results indicate that as expected the dentine enamel junction carries most of the occlusal morphology, however the enamel layer influences at least the blunting of the curvature of the dental topography, which may be associated with dietary specializations. Further studies on fossil lineages could potentially reveal that convergent emergences of similar occlusal topographies occurred following differential responses (or different selective mechanisms) of the enamel-dentine junction and enamel layer. Hence, the degree of independency of OES and EDJ morphologies is supposed to reflect the way the tooth morphotype is selected. In evo-devo analyses, our methods could not only help to determine whether changes implied by mutant genes influence the enamel dentine junction, the enamel layer or both (for example, variations of cusp slopes have been observed in knockout mice, e.g. [85], and have to be characterized), but could also bring localized quantifications of these changes, to quantify the rate of morphological change depending on the gene expression. On a theoretical level, it will be possible to perform covariation analyses to test whether there is an influence of the morphology of the enamel-dentine junction on the secretion of the enamel band and *vice versa*.

Our approach demonstrates to be a useful tool for characterizing and comparing tooth form and is therefore recommended as a component – possibly coupled with other methods such as geometric morphometrics or Occlusal Fingerprint Analysis (see e.g. [35], [39]) – in further dental studies. Besides, the developed analytical tools and procedures are suggested to apply to other tissues requiring a detailed tridimensional characterization.

## Supporting Information

Figure S1 **Surface alteration between original and decimated OES in two representative molars of** ***Homo*** **(A) and** ***Pan*** **(B).** The figure presents the alteration of the decimated surface as the distribution of the minimum distances from original to decimated polygon mesh. Maximum values (in millimeter) are +0.09 and −0.10 in *Homo* 5 (average +0.0036/−0.0039), and +0.083 and −0.055 for *Pan* 1 (average +0.058/−0.0057). Color scales indicate negative and positive distances from original to decimated surface (in millimeter). Although the reduction of polygon number may mask small morphological features, it prevents from documenting uninformative variation related to irregularity in individual polygon position and orientation. The decimation procedure does not change the overall shape of the tooth under consideration. The decimated surfaces correspond on average to 99% or the original area (e.g. 99.18% for *Homo* 5 and 99.08% for *Pan* 1). The decimation procedure typically affects the expression of crests and grooves (e.g. depth/elevation, see the case of chimpanzee and its highly crenulated occlusal enamel surface). However, the magnitude of morphological change remains low. While a lessened decimation procedure has to be considered for detailed studies of OES and EDJ, the present mesh resolution remains suitable for this note with reduced unwarranted noise and computational loads.
(TIF)

Figure S2 **Relative area of expression (mm^2^) of orientation intervals (increment is 45°) for enamel-dentine junction (EDJ) and enamel occlusal surfaces (OES).** A, *Lagothrix*; B, *Cercocebus*; C, *Cercopithecus*; D, *Papio*; E, *Hylobates*; F, *Gorilla*; G, *Pan*; H, *Homo*.
(TIF)

Figure S3 **Relationships between number of patches (complexity, ψ^c^) and 3D occlusal area.** A, OES, the star and the associated illustrated molar correspond to one chimpanzee specimen (P#2) showing a particularly high number of patch; B, OES, the specimen P#2 has been removed from the analysis; the star and the associated illustrated molar correspond to the second highest number of patch in chimpanzee. C, EDJ, note the variation in gorilla. For A, B, C, molar occlusal complexity (ψ^c^) is in abscissa and molar 3D occlusal area (mm^2^) in ordinate. D, OES: relationship between complexity computed on decimated surface (ψ^c/d^ this study, abscissa) and complexity computed on the full resolution surface (ψ^c^, ordinate). Note that higher resolution (i.e., increasing the number of polygon describing each occlusal surface) yields higher complexity values. E. EDJ: relationship between ψ^c/d^ (this study, abscissa) and ψ^c^ (ordinate).
(TIF)

Figure S4 **Relative contribution of the partial number of patches at each orientation interval to the total complexity.** The complete orientation range is divided in eight orientation intervals of 45° each, each color representing one particular orientation interval. Each ring corresponds to one specimen. The white dot indicates the highest computed (partial) complexity for a distribution. A, *Cercocebus*; *B*, *Cercopithecus*; C, *Papio*; D, *Lagothrix*; E, *Hylobates*; F1-F2, *Gorilla*; G1-G2, *Pan*; H1-H3, *Homo*.
(TIF)

Figure S5 **Inclination profiles of anthropoid molars.** The profile corresponds, for each taxon (average data), to the OES relative proportion of area of expression of inclination intervals (increment is 15°). Note how enamel deposit modifies the inclination profiles of EDJ (Figure 8).
(TIF)

Figure S6 **Occlusal relief index in anthropoids.** A, relationship between OES and EDJ occlusal relief index (Γ). B, comparison between occlusal relief index for OES (Γ^OES^) and *relief index* (RI, *sensu* Dennis et al., 2004; M'Kirera and Ungar, 2003, Ungar and Williamson 2000). Note the flattening of the RI profile compared to Γ^OES^. Dennis JC, Ungar, PS, Teaford M. F., Glander K. E. 2004. Dental Topography and Molar Wear in Alouatta palliate From Costa Rica. American Journal of Physical Anthropology 125: 152–161. M'Kirera F., Ungar P. 2003. Occlusal Relief Changes With Molar Wear in *Pan troglodytes troglodytes* and *Gorilla gorilla gorilla*. *American Journal of Primatology* 60: 31–41. Ungar P., Williamson M. 2000. Exploring the effects of toothwear on functional morphology: a preliminary study using dental topographic analysis. Palaeontologia Electronica, vol. 3: 1–18.
(TIF)

Figure S7 **Standardized mean curvature value of enamel-dentine junction surface.** The position of each taxon on the graph corresponds to the area proportion of enamel occlusal non-curved surface (left axe, horizontal line is for within taxon average value). The bars illustrate the associate proportion of area of expression of convex (light green)/concave (light orange) to highly/extremely convex (green) and highly concave (orange) surfaces (average value, see right panel).
(TIF)
