# Supplementary figures and images for: Prospective in (Primate) Dental Analysis through Tooth 3D Topographical Quantification
Source: PLoS One. 2013 Jun 24;8(6):e66142. doi: 10.1371/journal.pone.0066142 (PMC3691165; doi:10.1371/journal.pone.0066142)

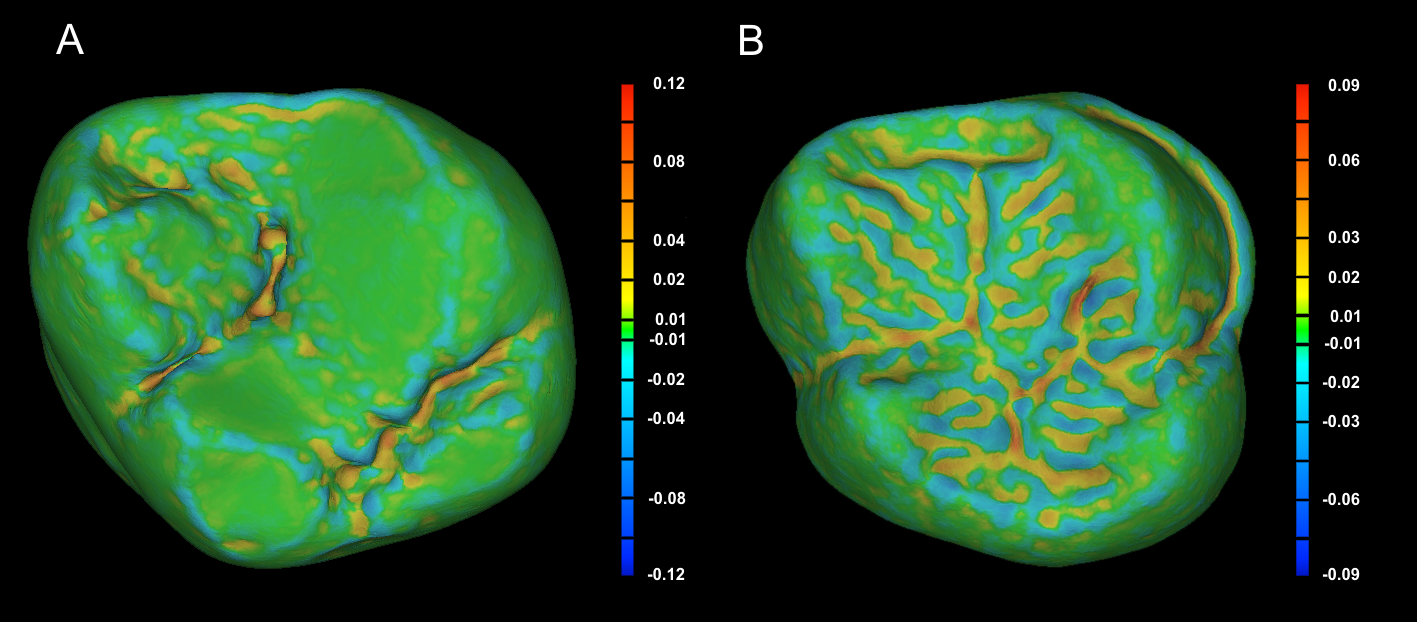

Supplement: Figure S1 — Surface alteration between original and decimated OES in two representative molars of Homo (A) and Pan (B). The figure presents the alteration of the decimated surface as the distribution of the minimum distances from original to decimated polygon mesh. Maximum values (in millimeter) are +0.09 and −0.10 in Homo 5 (average +0.0036/−0.0039), and +0.083 and −0.055 for Pan 1 (average +0.058/−0.0057). Color scales indicate negative and positive distances from original to decimated surface (in millimeter). Although the reduction of polygon number may mask small morphological features, it prevents from documenting uninformative variation related to irregularity in individual polygon position and orientation. The decimation procedure does not change the overall shape of the tooth under consideration. The decimated surfaces correspond on average to 99% or the original area (e.g. 99.18% for Homo 5 and 99.08% for Pan 1). The decimation procedure typically affects the expression of crests and grooves (e.g. depth/elevation, see the case of chimpanzee and its highly crenulated occlusal enamel surface). However, the magnitude of morphological change remains low. While a lessened decimation procedure has to be considered for detailed studies of OES and EDJ, the present mesh resolution remains suitable for this note with reduced unwarranted noise and computational loads. (TIF) [file pone.0066142.s001.tif]

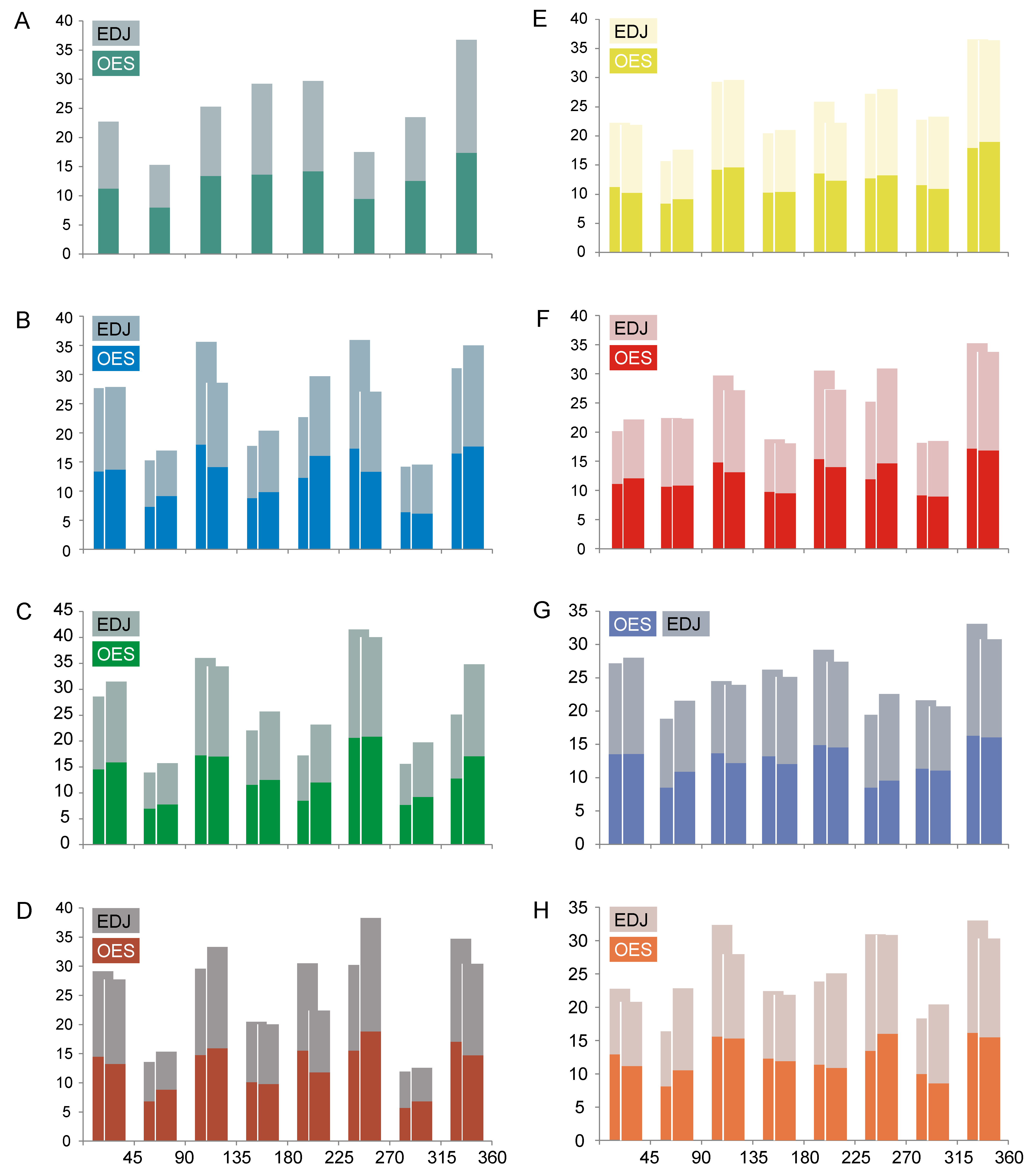

Supplement: Figure S2 — Relative area of expression (mm2) of orientation intervals (increment is 45°) for enamel-dentine junction (EDJ) and enamel occlusal surfaces (OES). A, Lagothrix; B, Cercocebus; C, Cercopithecus; D, Papio; E, Hylobates; F, Gorilla; G, Pan; H, Homo. (TIF) [file pone.0066142.s002.tif]

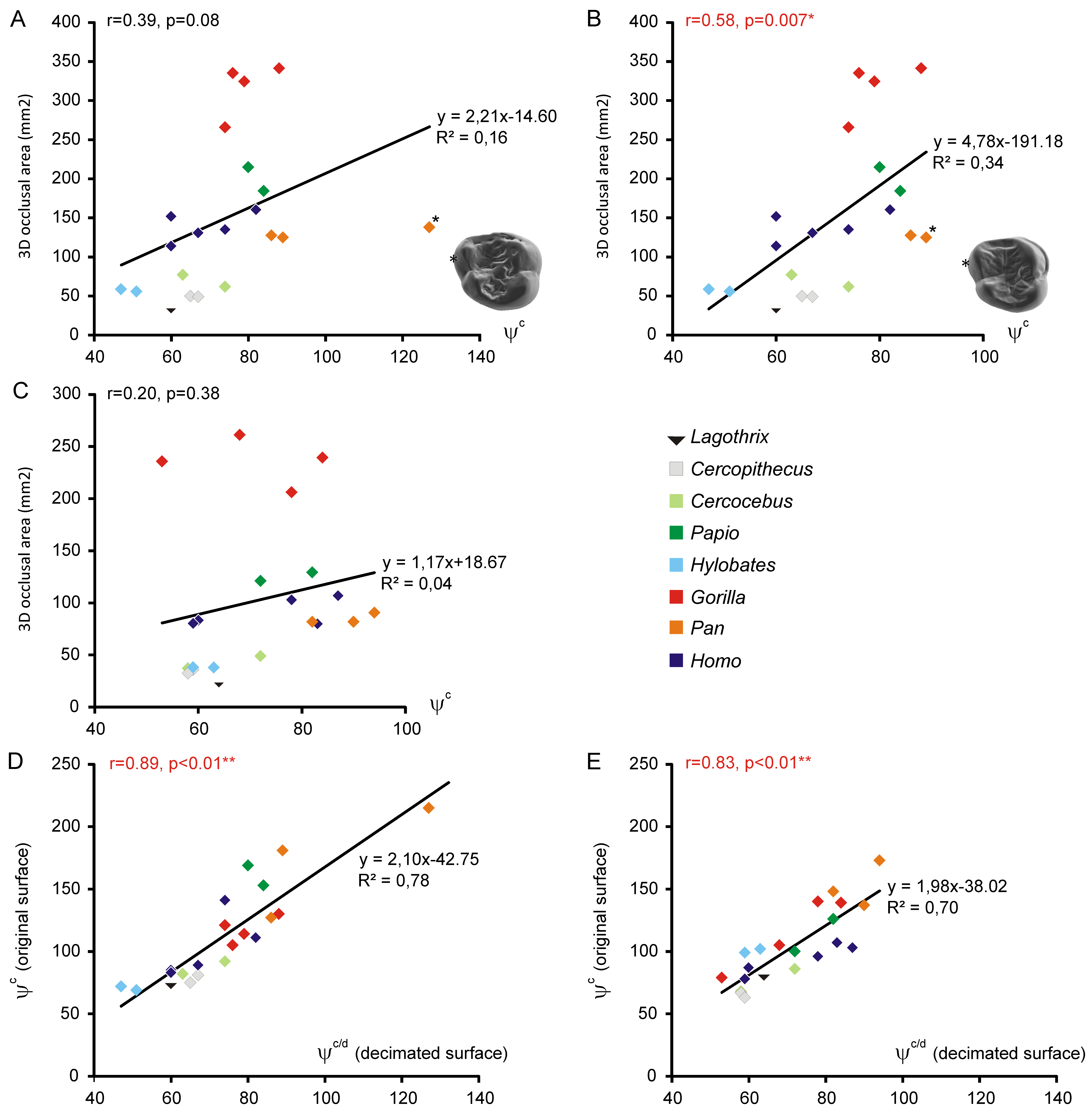

Supplement: Figure S3 — Relationships between number of patches (complexity, ψc) and 3D occlusal area. A, OES, the star and the associated illustrated molar correspond to one chimpanzee specimen (P#2) showing a particularly high number of patch; B, OES, the specimen P#2 has been removed from the analysis; the star and the associated illustrated molar correspond to the second highest number of patch in chimpanzee. C, EDJ, note the variation in gorilla. For A, B, C, molar occlusal complexity (ψc) is in abscissa and molar 3D occlusal area (mm2) in ordinate. D, OES: relationship between complexity computed on decimated surface (ψc/d this study, abscissa) and complexity computed on the full resolution surface (ψc, ordinate). Note that higher resolution (i.e., increasing the number of polygon describing each occlusal surface) yields higher complexity values. E. EDJ: relationship between ψc/d (this study, abscissa) and ψc (ordinate). (TIF) [file pone.0066142.s003.tif]

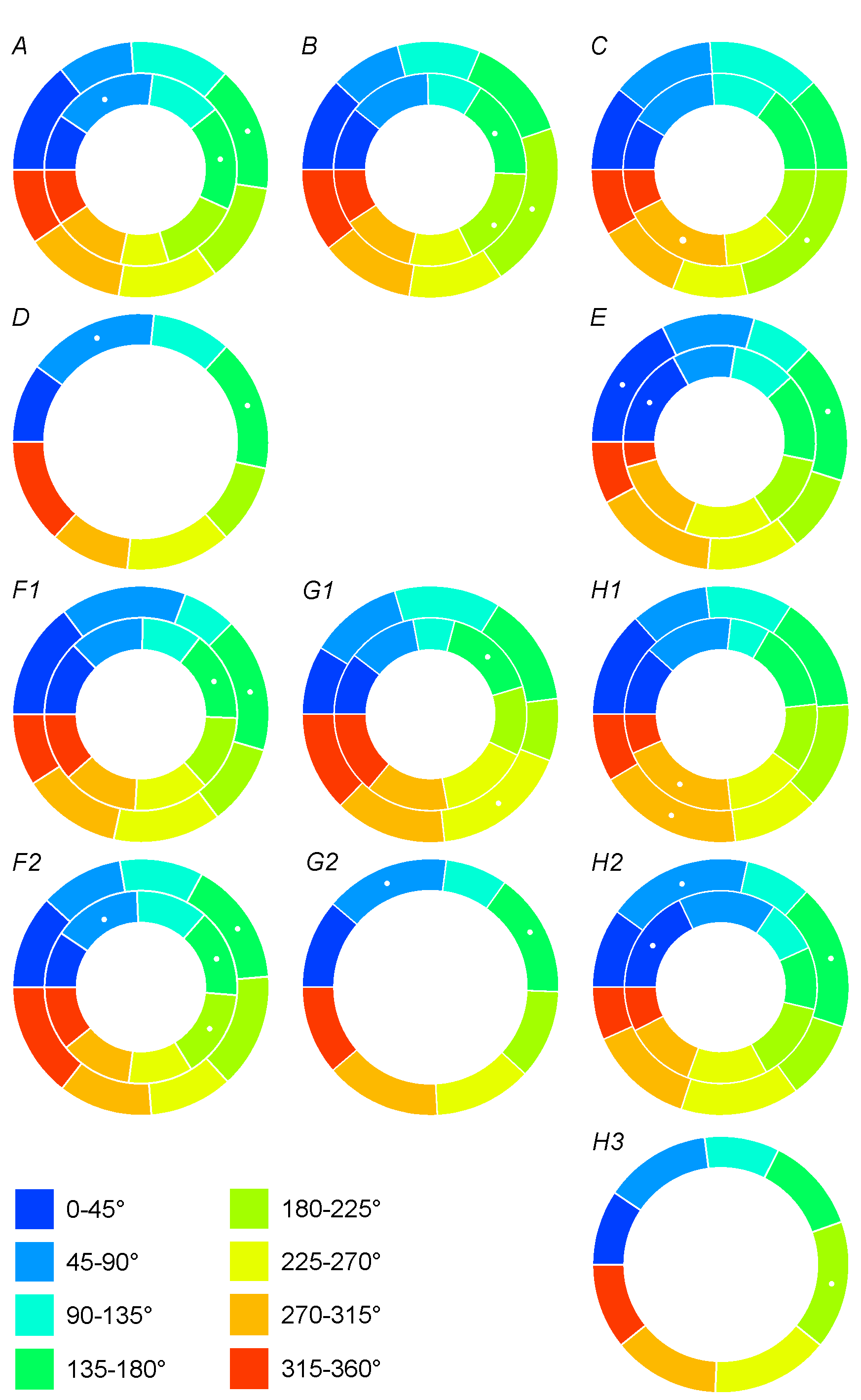

Supplement: Figure S4 — Relative contribution of the partial number of patches at each orientation interval to the total complexity. The complete orientation range is divided in eight orientation intervals of 45° each, each color representing one particular orientation interval. Each ring corresponds to one specimen. The white dot indicates the highest computed (partial) complexity for a distribution. A, Cercocebus; B, Cercopithecus; C, Papio; D, Lagothrix; E, Hylobates; F1-F2, Gorilla; G1-G2, Pan; H1-H3, Homo. (TIF) [file pone.0066142.s004.tif]

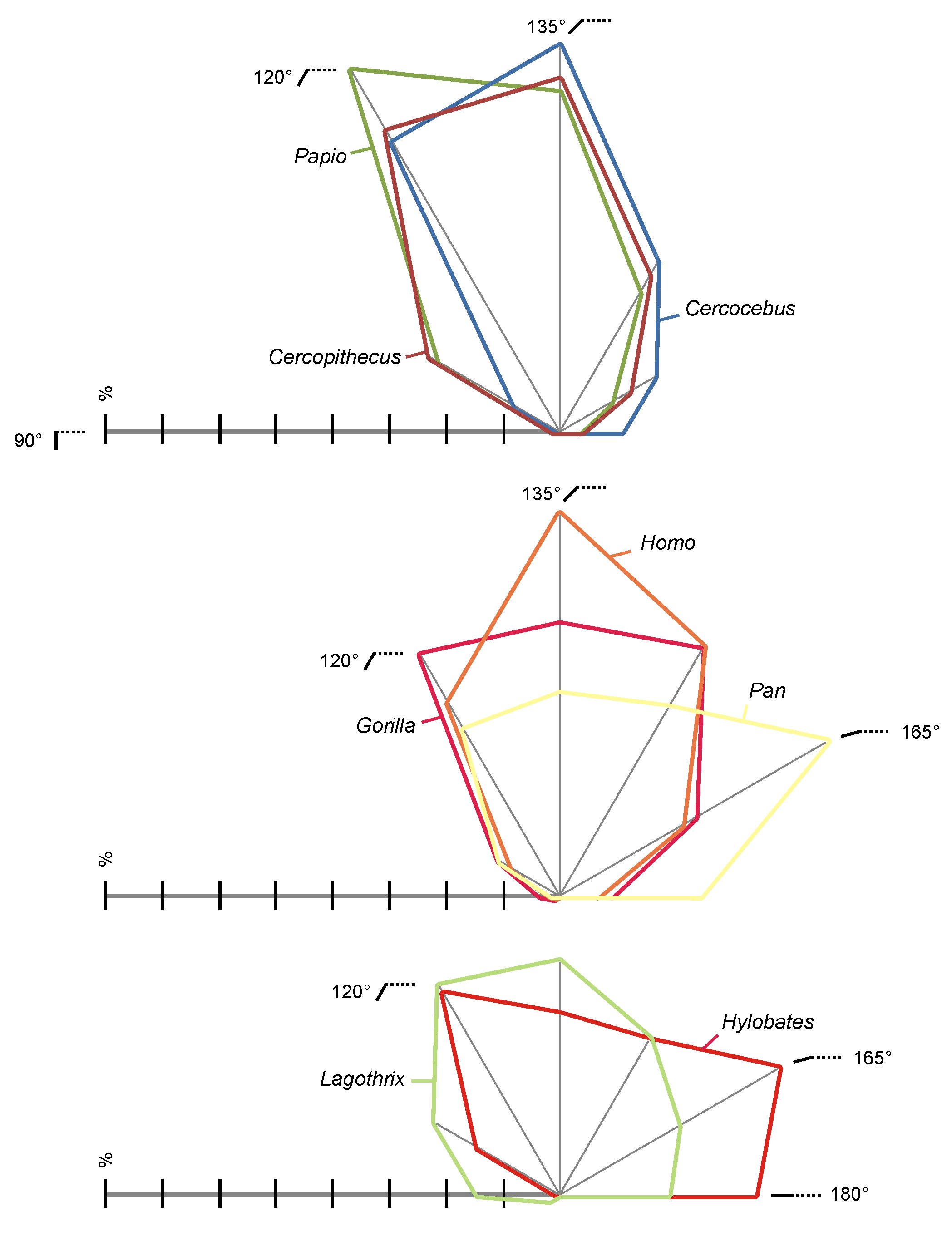

Supplement: Figure S5 — Inclination profiles of anthropoid molars. The profile corresponds, for each taxon (average data), to the OES relative proportion of area of expression of inclination intervals (increment is 15°). Note how enamel deposit modifies the inclination profiles of EDJ (Figure 8). (TIF) [file pone.0066142.s005.tif]

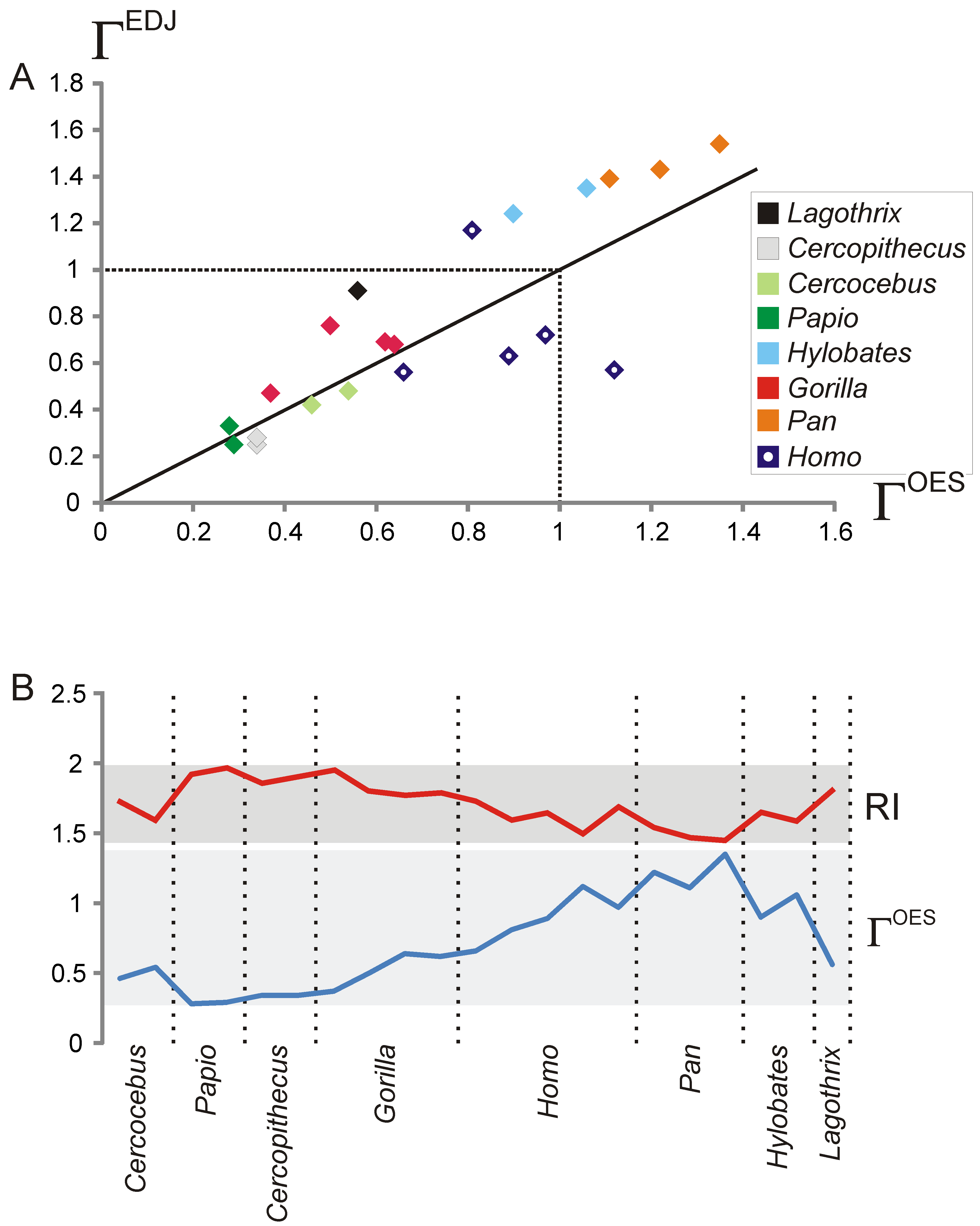

Supplement: Figure S6 — Occlusal relief index in anthropoids. A, relationship between OES and EDJ occlusal relief index (Γ). B, comparison between occlusal relief index for OES (ΓOES) and relief index (RI, sensu Dennis et al., 2004; M'Kirera and Ungar, 2003, Ungar and Williamson 2000). Note the flattening of the RI profile compared to ΓOES. Dennis JC, Ungar, PS, Teaford M. F., Glander K. E. 2004. Dental Topography and Molar Wear in Alouatta palliate From Costa Rica. American Journal of Physical Anthropology 125: 152–161. M'Kirera F., Ungar P. 2003. Occlusal Relief Changes With Molar Wear in Pan troglodytes troglodytes and Gorilla gorilla gorilla. American Journal of Primatology 60: 31–41. Ungar P., Williamson M. 2000. Exploring the effects of toothwear on functional morphology: a preliminary study using dental topographic analysis. Palaeontologia Electronica, vol. 3: 1–18. (TIF) [file pone.0066142.s006.tif]

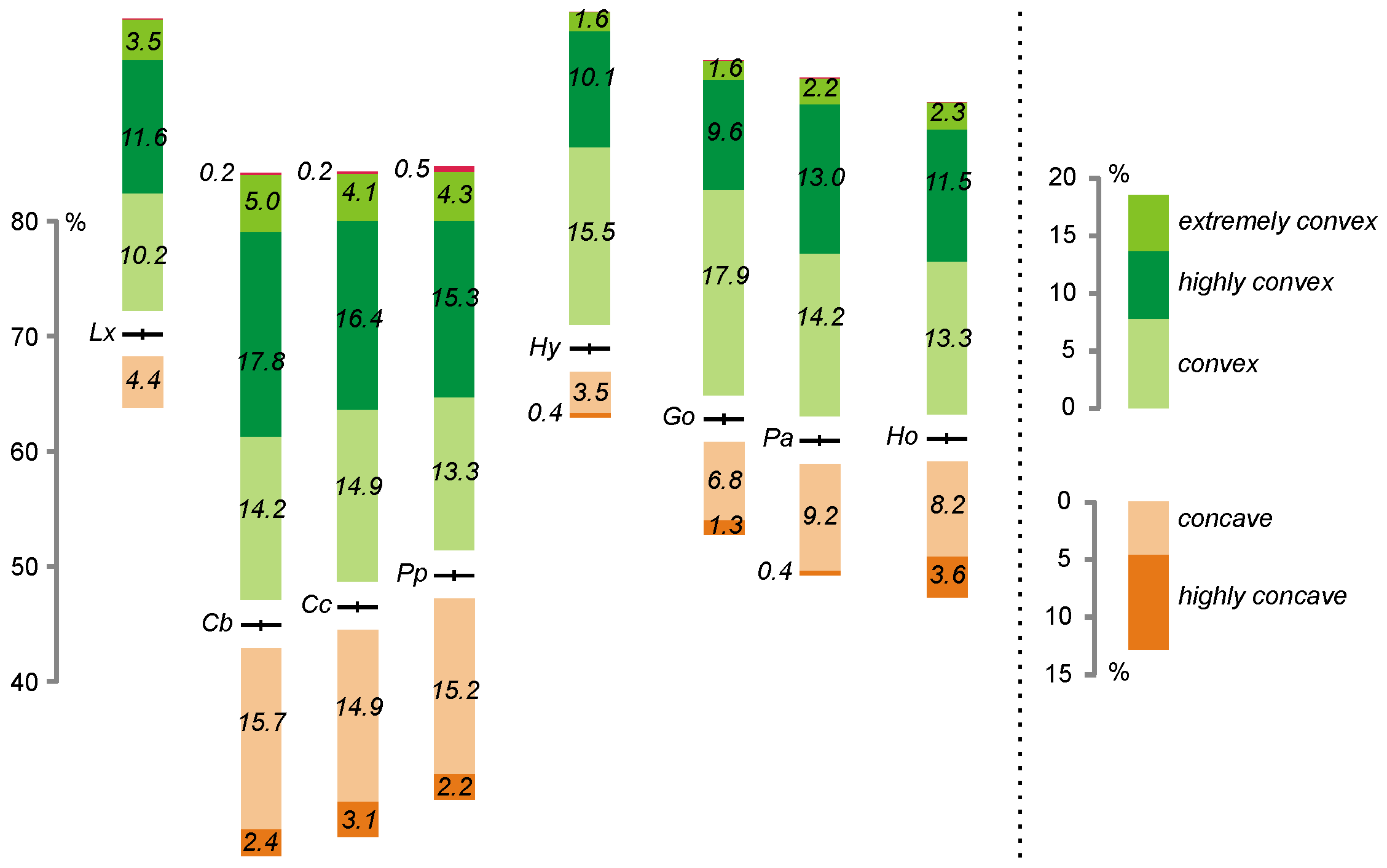

Supplement: Figure S7 — Standardized mean curvature value of enamel-dentine junction surface. The position of each taxon on the graph corresponds to the area proportion of enamel occlusal non-curved surface (left axe, horizontal line is for within taxon average value). The bars illustrate the associate proportion of area of expression of convex (light green)/concave (light orange) to highly/extremely convex (green) and highly concave (orange) surfaces (average value, see right panel). (TIF) [file pone.0066142.s007.tif]
